# Levetiracetam therapeutically targets GABAergic synapses in diffuse midline glioma

**DOI:** 10.1038/s41591-026-04646-6

**Published:** 2026-09-17

**Authors:** Tara Barron, Richard Drexler, Aaron Mochizuki, Evan Cantor, Kiarash Shamardani, Pamelyn J. Woo, Neeraj Soni, Dana Tlais, Andrea Franson, Samantha Lyons, Truman Knowles, Shannon Raber, Vilina Mehta, Michael A. Quezada, Michael B. Keough, Samin Malaki Jahan, Linh Thuy Vo, Enrique Herrera Castañeda, Kathryn R. Taylor, Lijun Ni, Kim Kilian, Samuel H. Wu, Franz L. Ricklefs, Ulrich Schüller, Sabine Mueller, Paul G. Fisher, Cynthia J. Campen, Hannes Vogel, Sonia Partap, Kai Kaila, Carl Koschmann, Michelle Monje

**Affiliations:** 1 Department of Neurology and Neurological Sciences, Stanford University, Stanford, CA USA; 2 Department of Pathology, University of Texas Southwestern Medical Center, Dallas, TX USA; 3 Harold C. Simmons Comprehensive Cancer Center, Dallas, TX USA; 4 Peter O’Donnell Jr. Brain Institute, UT Southwestern Medical Center, Dallas, TX USA; 5 Department of Pediatric Hematology/Oncology, University of Michigan, Ann Arbor, MI USA; 6 University of California, San Francisco, San Francisco, CA USA; 7 Department of Neurosurgery, University Medical Center Hamburg-Eppendorf, Hamburg, Germany; 8 Institute of Neuropathology, University Medical Center Hamburg-Eppendorf, Hamburg, Germany; 9 Department of Pediatric Hematology and Oncology, University Medical Center Hamburg-Eppendorf, Hamburg, Germany; 10 Research Institute Children’s Cancer Center, Hamburg, Germany; 11 Department of Pathology, Stanford University, Stanford, CA USA; 12 Faculty of Bio- and Environmental Sciences (MIBS), University of Helsinki, Helsinki, Finland; 13 Howard Hughes Medical Institute, Stanford University, Stanford, CA USA; 14 Department of Neurosurgery, Stanford University, Stanford, CA USA; 15 Department of Pediatrics, Stanford University, Stanford, CA USA

**Keywords:** Cancer in the nervous system, CNS cancer

## Abstract

Antiseizure medications, such as levetiracetam, are commonly used in glioma patients. Retrospective analyses evaluating effects of antiseizure medications on glioma patient survival have yielded inconsistent results, probably due to confounding factors of glioma subtype and drug class. Here we present retrospective real-world clinical data that demonstrate longer overall survival for children with diffuse midline glioma (DMG) who were taking levetiracetam, which was not evident in pediatric patients with hemispheric high-grade glioma. In preclinical models, levetiracetam reduces glioma proliferation and tumor burden, extending survival of mice bearing DMG orthotopic xenografts. These beneficial effects were not found in hemispheric high-grade glioma patient-derived orthotopic xenograft models. The subtype-specificity of this antiproliferative effect of levetiracetam is congruent with recent findings that GABAergic neuron-to-glioma synapses promote glioma growth in DMG but not hemispheric high-grade glioma. We demonstrate here that levetiracetam attenuates low-frequency GABAergic synaptic transmission in a glioma-specific manner, reducing GABAergic synaptic currents in DMG but not in healthy neurons. This effect is independent of action on SV2A, the chief mechanism by which levetiracetam functions to prevent and treat seizures. Taken together, these findings indicate a promising approach to target GABAergic synapses in DMG and suggest that use of levetiracetam in DMG should be further studied in future prospective clinical studies.

## Abstract (web summary)

Results of this study show in experimental models and data from patient cohorts that the antiseizure medication levetiracetam is associated with longer survival and reduced tumor growth in diffuse midline glioma, but not hemispheric high-grade glioma, by selectively dampening GABAergic synaptic signaling, independently of its canonical SV2A-mediated primary antiseizure mechanism.

## Main

High-grade gliomas (HGGs) are the leading cause of brain tumor-related morbidity and mortality in children. Pediatric-type gliomas are broadly classified^1^ as hemispheric HGGs that occur in the cerebral hemispheres and diffuse midline gliomas (DMGs) that occur in midline central nervous system structures, most commonly the brainstem, thalamus or spinal cord, and are often associated with H3K27M mutations in genes encoding histone H3.

Neurons robustly drive the growth and progression of gliomas in a neuronal activity-regulated manner^2^ through both activity-regulated paracrine factor signaling^2–7^ and bona fide, electrophysiologically functional neuron-to-glioma synapses^6,8–11^. Glioma cell membrane depolarization, through excitatory postsynaptic currents or other mechanisms, promotes glioma cell proliferation and tumor growth^6,8^ via voltage-sensitive mechanisms that remain to be fully elucidated. Just as neuronal activity promotes glioma growth and progression, gliomas increase neuronal excitability^8,12–16^. This glioma-induced neuronal hyperexcitability promotes glioma-associated seizures^12–15^ and further augments this pathogenic cycle of neuron–glioma interactions.

So far, glutamatergic neurons have been most extensively studied in this regard, and glutamatergic neuron-to-glioma synapses mediated by AMPA glutamate receptors are present in both DMG and hemispheric HGGs^8,9^. GABAergic neuron-to-glioma synapses are found in DMG where GABAergic synaptic signaling is depolarizing due to high intracellular chloride concentration in DMG malignant cells^11^. GABAergic neurons powerfully promote DMG growth, and medications that augment GABAergic signaling—for example, benzodiazepines such as lorazepam—accelerate DMG growth and shorten survival in patient-derived orthotopic xenograft models^11^. By contrast, GABA evokes only minimal currents in the hemispheric HGG models studied so far^11^, and positive allosteric modulators of GABAergic signaling had no effect in these preclinical models of hemispheric HGG^11^. Thus, depolarizing GABAergic neuron-to-glioma synapses appear to be more enriched in DMGs, highlighting the concept of tumor subtype-specific neurophysiology^11^.

These insights regarding neuron–glioma interactions shine a light on a long-standing clinical question of whether to recommend antiseizure medication usage in patients with glioma who have not yet had clinically apparent seizures. Retrospective analyses including a large collaborative meta-analysis of the correlation between antiseizure medication usage and survival outcomes have not found consistent evidence of antiseizure medication benefit in patients with brain tumors^17^. However, these studies have chiefly focused on adult patients, include several distinct brain cancer types and often include multiple different antiseizure medications. Some studies have focused on a very commonly used antiseizure medication with a favorable side effect profile called levetiracetam in patients with glioma, and these previous studies have reported conflicting findings about effects of levetiracetam on glioma—chiefly adult glioblastoma—patient outcomes^18–20^. Hypothesizing that the tumor subtype-specific neurophysiology of DMG may predict tumor subtype-specific effects of levetiracetam in pediatric patients with HGGs, we studied levetiracetam in patients and preclinical models of DMG.

## Results

### Levetiracetam is associated with extended survival in retrospective data of patients with DMG

**Fig. 1:**
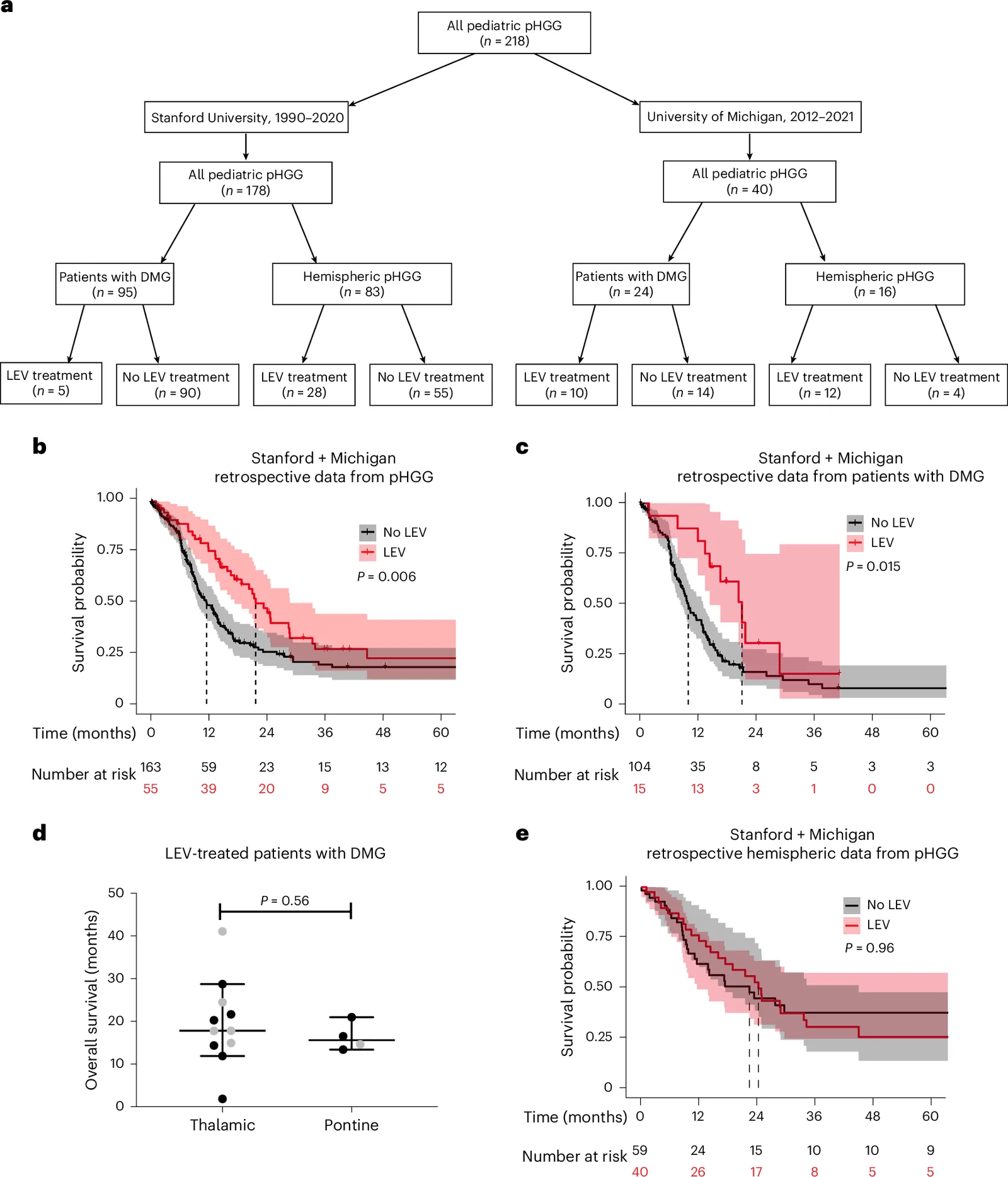
LEV extends OS of patients with DMG in retrospective, real-world patient data. **a**, An overview of patients included in the primary cohort from Stanford University and University of Michigan. **b**, All pediatric HGGs: Kaplan–Meier OS curves of retrospective data from Stanford University (1990–2020) and University of Michigan (2012–2021) patient databases (*n* = 218), showing pediatric patients with HGG (pHGG) treated with levetiracetam (LEV) (LEV: median OS of 21.6 months, *n* = 55; no LEV: median OS of 11.5 months, *n* = 163), two-tailed log-rank test. **c**, DMGs: Kaplan–Meier OS curves of retrospective data from patients with DMG treated with LEV (LEV: median OS of 20.96 months, *n* = 15; no LEV: median OS of 9.92 months, *n* = 104), two-tailed log-rank test. **d**, No difference in OS of LEV-treated patients with thalamic compared to pontine DMG (thalamic: *n* = 11; pontine: *n* = 4). The gray points are data censored at time of last follow-up, two-tailed Student’s *t*-test. **e**, Hemispheric HGGs: Kaplan–Meier OS curves of retrospective data from patients with hemispheric HGG treated with LEV (LEV: OS of 24.0 months, *n* = 40; no LEV: OS of 22.2 months, *n* = 59), two-tailed log-rank test. Data are median ± 95% confidence interval. Source data

Here, we assessed retrospective, real-world data from two major US pediatric neuro-oncology centers (Stanford University and University of Michigan) to query possible effects of levetiracetam on overall survival (OS) in pediatric patients with HGGs (Fig. 1a). For all DMG cases with available tissue, central pathology review was performed by a board-certified neuropathologist (H.V.) to confirm diagnosis of DMG. The patient cohorts examined predated introduction of CAR T cell therapy for DMG. Kaplan–Meier analysis of all pediatric HGG patients (*n* = 218) suggests a survival advantage of levetiracetam usage (Fig. 1b). For multivariable survival analysis, we utilized an elastic net-regularized Cox regression for variable selection and found that in all patients with HGG, a diagnosis of DMG was—as expected—associated with decreased OS (coefficient of +0.55) and that thalamic DMG tumor location (coefficient of −0.20) and levetiracetam (coefficient of −0.11) were associated with increased OS. The variables of age, sex, ONC201 usage and panobinostat usage had coefficients of zero. Conventional or targeted chemotherapy other than ONC201 and panobinostat were surprisingly associated with increased OS (coefficient of −0.61); this may be explained by the observation that pontine DMG subjects in the historical database often did not receive any conventional or targeted therapy (Supplementary Table 1) due to demonstrated lack of efficacy of conventional chemotherapy in pontine DMG^21^, and thus, conventional chemotherapy usage may be a surrogate marker of glioma type in this dataset. Hypothesizing that DMGs drove the positive survival association of levetiracetam usage, we next evaluated DMG and hemispheric HGGs separately. These databases include subjects with biopsy-demonstrated H3K27M-mutated or H3 wild-type (WT) DMGs as well as subjects before availability of molecular testing for whom diagnosis was based only on the typical radiographic appearance of DMGs; both H3K27M-altered and H3 WT subgroups of DMGs are therefore included (Supplementary Table 1). The DMG analysis suggests that patients with DMG (*n* = 119) who had a history of levetiracetam usage (*n* = 15 children) exhibited a longer median OS compared to those without levetiracetam usage (*n* = 104 children) (Fig. 1c and Supplementary Table 1). Those patients with DMG with a history of levetiracetam usage had a median OS of 20.96 months, compared to those without levetiracetam usage who exhibited a median OS of 9.92 months (*P* = 0.015). Of note, thalamic DMG represented a higher proportion of the group with a history of levetiracetam usage than the group with no history of levetiracetam usage. Comparing subjects with thalamic and pontine DMG who received levetiracetam, we find no difference in OS in this levetiracetam usage group (Fig. 1d), suggesting that the higher proportion of thalamic DMG in this group does not account for the observed increased median OS compared to the group without levetiracetam usage. The median OS of the control (no levetiracetam usage) group is consistent with the expected median OS for diffuse intrinsic pontine glioma (DIPG)/DMG (10–11 months for pontine DMG, 13 months for thalamic DMG)^22,23^. Analysis of the retrospective clinical data focused on hemispheric pediatric HGGs revealed no effect of levetiracetam usage on OS in pediatric patients with non-DMG, hemispheric HGGs (*n* = 40 patients with levetiracetam use, median OS of 24.0 months versus *n* = 59 patients without levetiracetam use, median OS of 22.2 months, *P* = 0.96) (Fig. 1e and Supplementary Table 2).

**Fig. 2:**
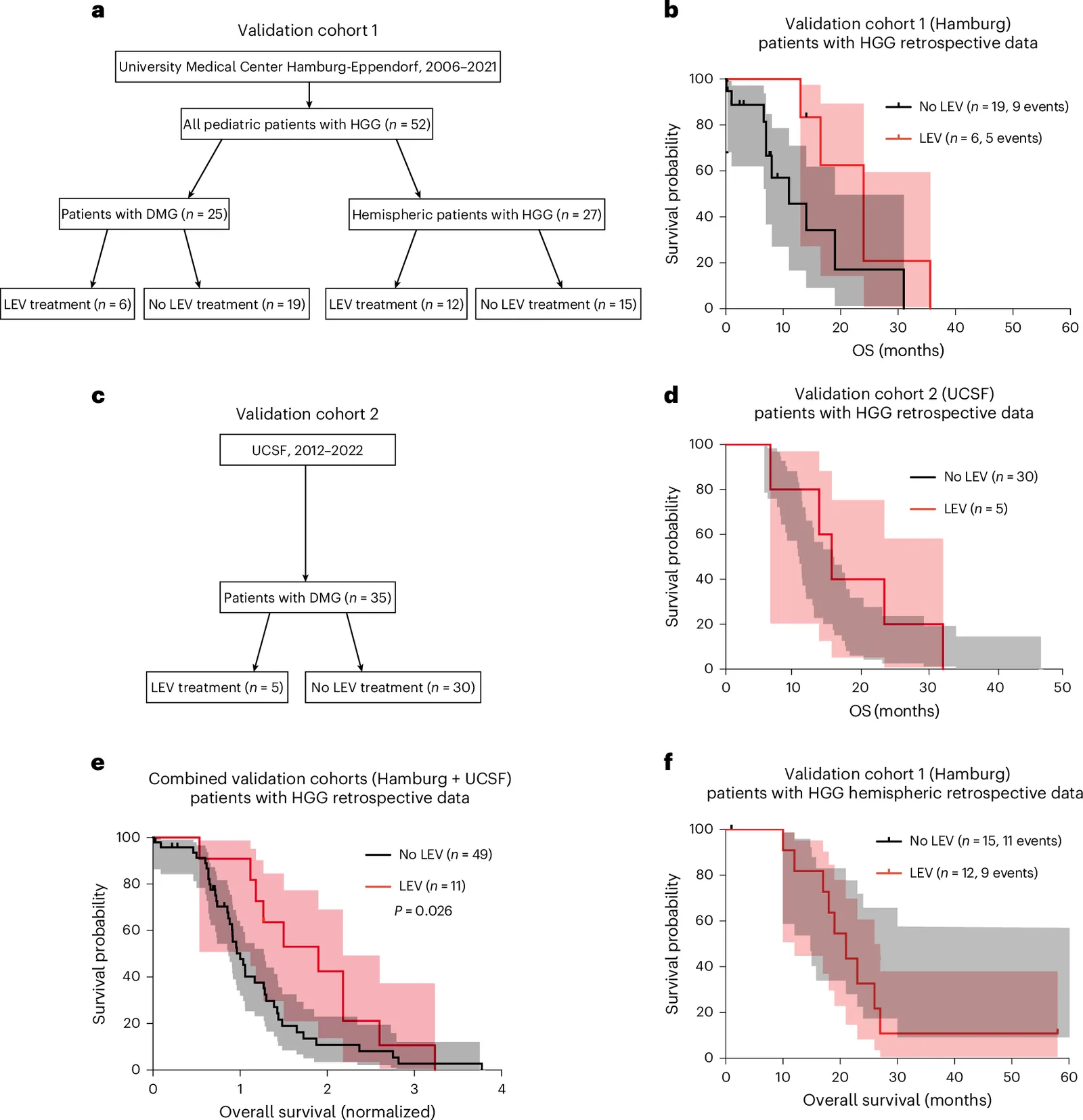
Validation of retrospective patient data. **a**, Overview of patients included in validation cohort 1 from University Medical Center Hamburg-Eppendorf. **b**, Validation cohort 1, from University Medical Center Hamburg-Eppendorf, DMGs: Kaplan–Meier OS curves of retrospective data from patients with DMGs treated with levetiracetam (LEV) (LEV: median OS of 24.0 months, *n* = 6; no LEV: median OS of 11.0 months, *n* = 19). **c**, Overview of patients included in validation cohort 2 from UCSF. Only patients who were not on a clinical trial were included. **d**, Validation cohort 2, from UCSF, DMGs: Kaplan–Meier OS curves of retrospective data from DMG patients treated with LEV (LEV: median OS of 15.5 months, *n* = 5; no LEV: median OS of 12.2 months, *n* = 30). **e**, Combined validation cohorts, DMGs: Kaplan–Meier OS curves of retrospective data from patients with DMG treated with LEV, normalized to median of no LEV control for each dataset (LEV: normalized median of 1.89785, *n* = 11; no LEV: normalized median of 1, *n* = 49), two-tailed Gehan–Breslow–Wilcoxon test. **f**, Validation cohort 1, hemispheric HGG: Kaplan–Meier OS curves of retrospective data from patients with hemispheric HGG treated with LEV (LEV: median OS of 21.0 months, *n* = 12; no LEV: median OS of 23.5 months, *n* = 15), two-tailed log-rank test. Data are median ± 95% confidence interval. Source data

To further explore the relationship between levetiracetam usage and DMG outcomes, we examined retrospective validation cohorts from two additional institutions, each of which showed the same trend of longer survival in patients treated with levetiracetam. The first validation cohort (from Hamburg Hospital, Germany; Fig. 2a), indicated a longer median OS for patients with DMG who had a history of levetiracetam usage compared to those without levetiracetam usage (*n* = 6 children with levetiracetam, median OS of 24.0 months versus *n* = 19 children without levetiracetam use, median OS of 11.0 months; Fig. 2b). The second validation cohort of 35 patients with DMG (from University of California, San Francisco (UCSF); only patients who were not on a clinical trial were included; Fig. 2c), indicated the same trend (*n* = 5 children with DMG that had been treated with levetiracetam (median OS of 15.5 months) and *n* = 30 children without levetiracetam use (median OS of 12.2 months)) (Fig. 2d). We combined the data from both institutions normalized to the median of the control group for each dataset. This combined validation cohort (Hamburg + UCSF) supported the conclusion that there was a survival benefit in patients treated with levetiracetam (*n* = 11 children with versus *n* = 49 children without levetiracetam use; Fig. 2e). In the Hamburg dataset, children with hemispheric HGGs were also available for analysis; as with the Stanford–Michigan dataset described above, there was no difference in survival in pediatric patients with hemispheric HGG with or without a history of levetiracetam treatment (*n* = 12 children, median OS of 21.0 months versus *n* = 15 children, median OS of 23.5 months with and without levetiracetam use, respectively; Fig. 2f).

Taken together, these real-world data across four independent institutions suggest a possible survival benefit of levetiracetam specifically in patients with DMG. Important caveats are that these data are retrospective, the numbers are small and levetiracetam should be studied in future prospective clinical studies with stratification by molecular subtype and DMG location before drawing conclusions.

### Levetiracetam reduces glioma growth and extends survival in preclinical models of DMG

**Fig. 3:**
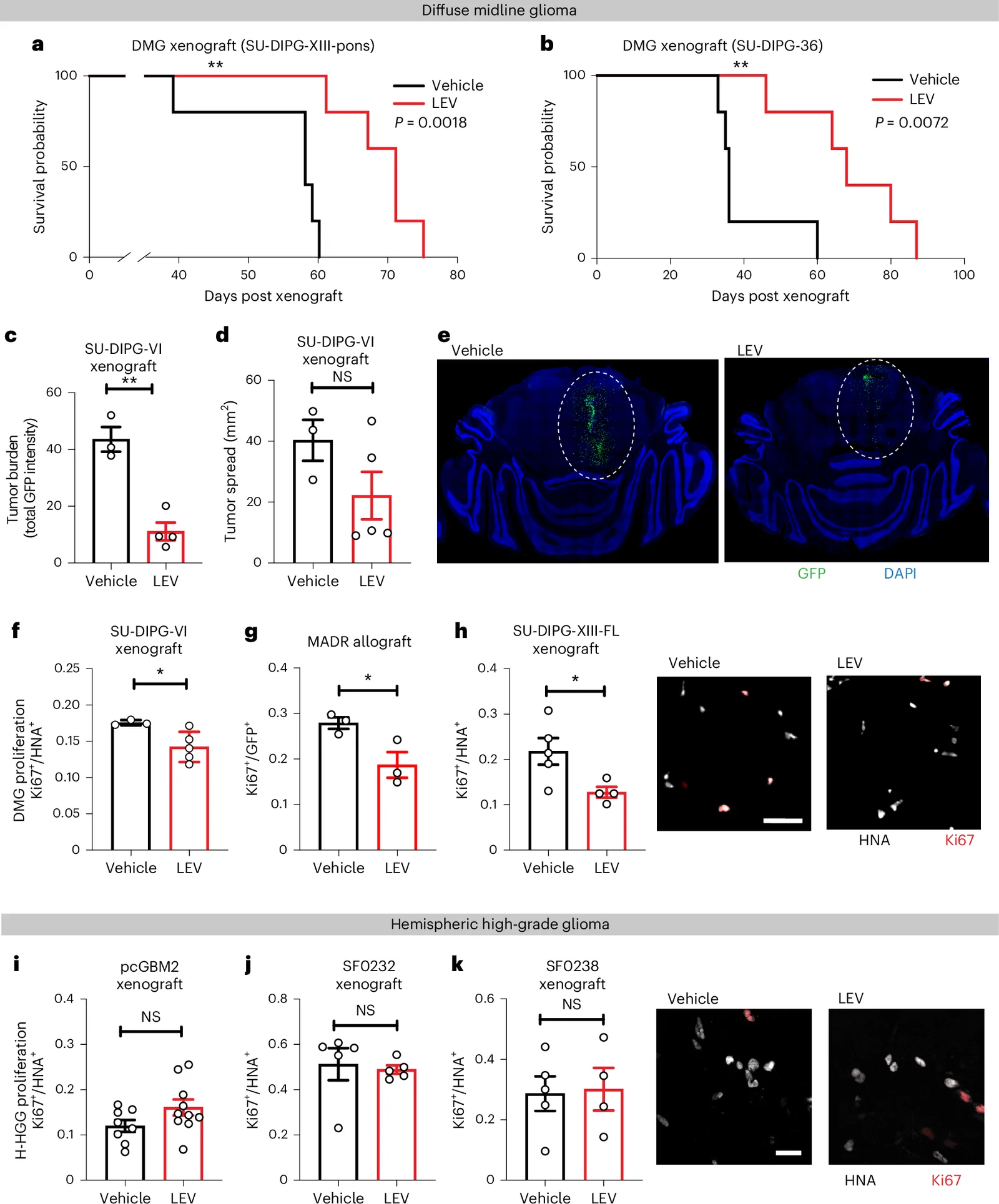
LEV extends survival and reduces glioma growth in DMG but not hemispheric HGG preclinical models. **a**,**b**, Kaplan–Meier survival curves of mice with orthotopically xenografted SU-DIPG-XIII-pons (**a**) or SU-DIPG-36 (**b**) patient-derived DMG cells treated with levetiracetam (LEV) or vehicle (*n* = 5 mice per group). **c**,**d**, Effect of LEV on overall tumor burden (**c**) and tumor spread (**d**) in mice with patient-derived DMG xenografts (SU-DIPG-VI; vehicle, *n* = 3; LEV, *n* = 5), two-tailed Student’s *t*-test. **e**, Representative confocal images of xenografted SU-DIPG-VI cells (green) in the pons of mice treated with vehicle or LEV. The dashed white line represents area of tumor spread. **f**–**h**, Effect of LEV treatment on glioma proliferation in mice with patient-derived DMG xenografts or syngeneic allografts: SU-DIPG-VI xenografts (vehicle, *n* = 3 mice; LEV, *n* = 5 mice; *P* = 0.0370) (**f**), MADR allografts (vehicle, *n* = 3 mice; LEV, *n* = 3 mice; *P* = 0.0411) (**g**), SU-DIPG-XIII-FL xenografts (vehicle, *n* = 5 mice; LEV, *n* = 4 mice; *P* = 0.0363) (**h**), two-tailed Student’s *t*-test. Right: representative confocal images of xenografted SU-DIPG-XIII-FL cells expressing Ki67 (red) and HNA (white). Scale bar, 25 µm. **i**–**k**, No effect of LEV treatment on glioma proliferation in mice with patient-derived IDH WT/H3 WT hemispheric HGG (H-HGG) xenografts, SU-pcGBM2 (vehicle, *n* = 8 mice; LEV, *n* = 10 mice) (**i**), SF0232 (vehicle, *n* = 5 mice; LEV, *n* = 5 mice) (**j**) and SF0238 (vehicle, *n* = 5 mice; LEV, *n* = 4 mice) (**k**), two-tailed Student’s *t*-test. Right: representative confocal images of xenografted SF0238 cells expressing Ki67 (red) and HNA (white). Scale bar, 25 µm. For all experiments, LEV (20 mg kg^−1^) was administered for 4 weeks before analyses. All data are mean ± s.e.m. NS, not significant, *P < 0.05, **P < 0.01. Source data

**Fig. 4:**
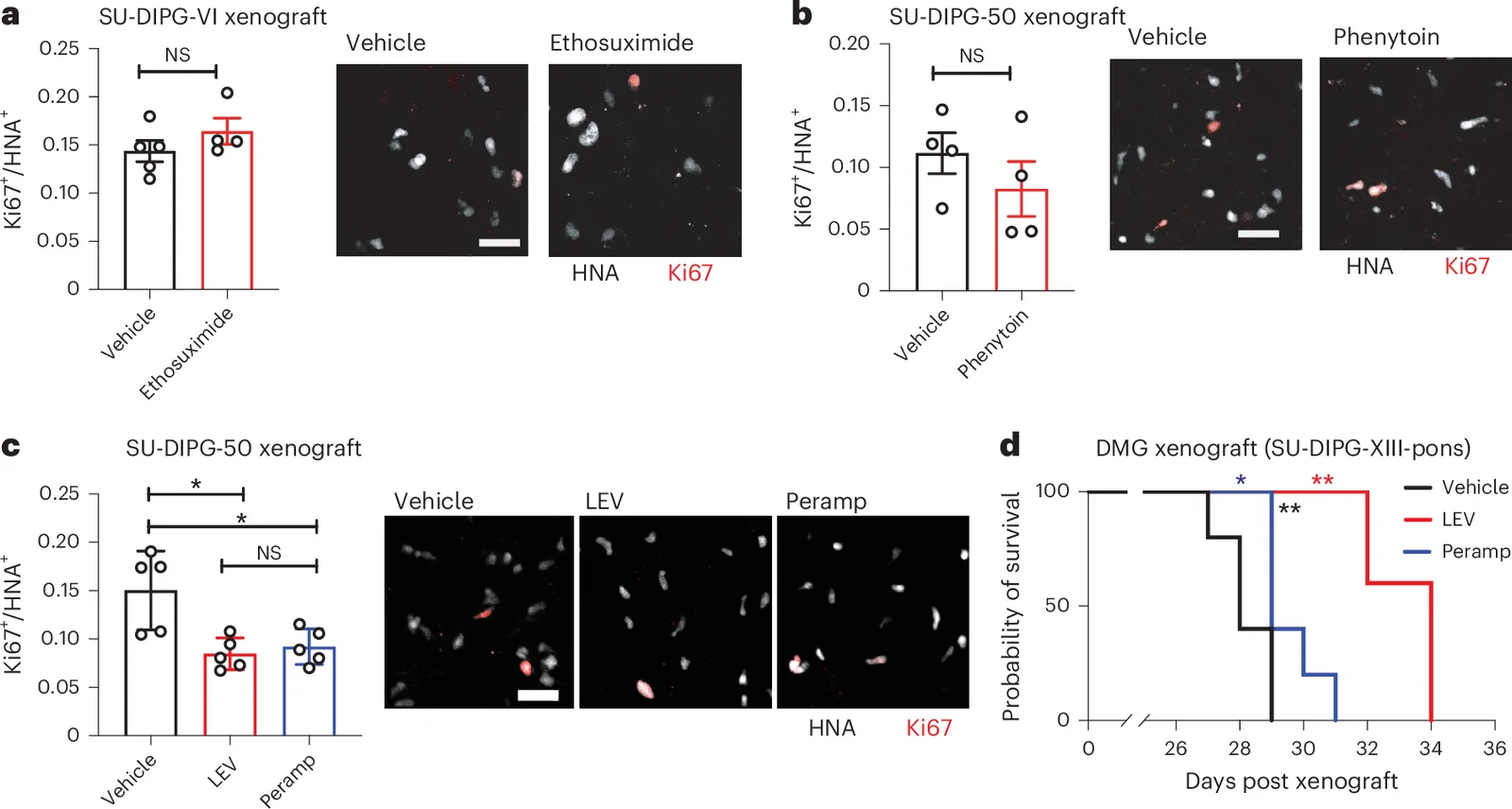
Antiseizure drugs have variable effects on DMG growth. **a**, Ethosuximide treatment in mice with patient-derived SU-DIPG-VI xenografts has no effect on cell proliferation (vehicle, *n* = 5 mice; ethosuximide, *n* = 4 mice). Right: representative confocal images of xenografted SU-DIPG-VI cells expressing Ki67 (red) and HNA (white). Scale bar, 25 µm. Two-tailed Student’s *t*-test. **b**, Phenytoin treatment in mice with patient-derived SU-DIPG-50 xenografts has no effect on cell proliferation (vehicle, *n* = 4 mice; phenytoin, *n* = 4 mice). Right: representative confocal images of xenografted SU-DIPG-50 cells expressing Ki67 (red) and HNA (white). Scale bar, 25 µm. Two-tailed Student’s *t*-test. **c**, Effect of levetiracetam (LEV) and perampanel (Peramp) treatment on glioma proliferation in mice with patient-derived DMG xenografts (SU-DIPG-50; *n* = 5 mice for each group; *P* = 0.0103 for vehicle versus LEV; *P* = 0.0196 for vehicle versus Peramp), two-tailed Student’s *t*-test. Right: representative confocal images of xenografted SU-DIPG-50 cells expressing Ki67 (red) and HNA (white). Scale bar, 25 µm. **d**. Kaplan–Meier survival curves of mice with orthotopically xenografted SU-DIPG-XIII-pons tumors treated with LEV, Peramp or vehicle (*n* = 5 mice per group; *P* = 0.0309 for vehicle versus Peramp, *P* = 0.0026 for vehicle versus LEV, *P* = 0.0015 for LEV versus Peramp). Two-tailed log-rank test. All data are mean ± s.e.m. NS, not significant, *P < 0.05, **P < 0.01. Source data

**Extended Data Fig. 1:**
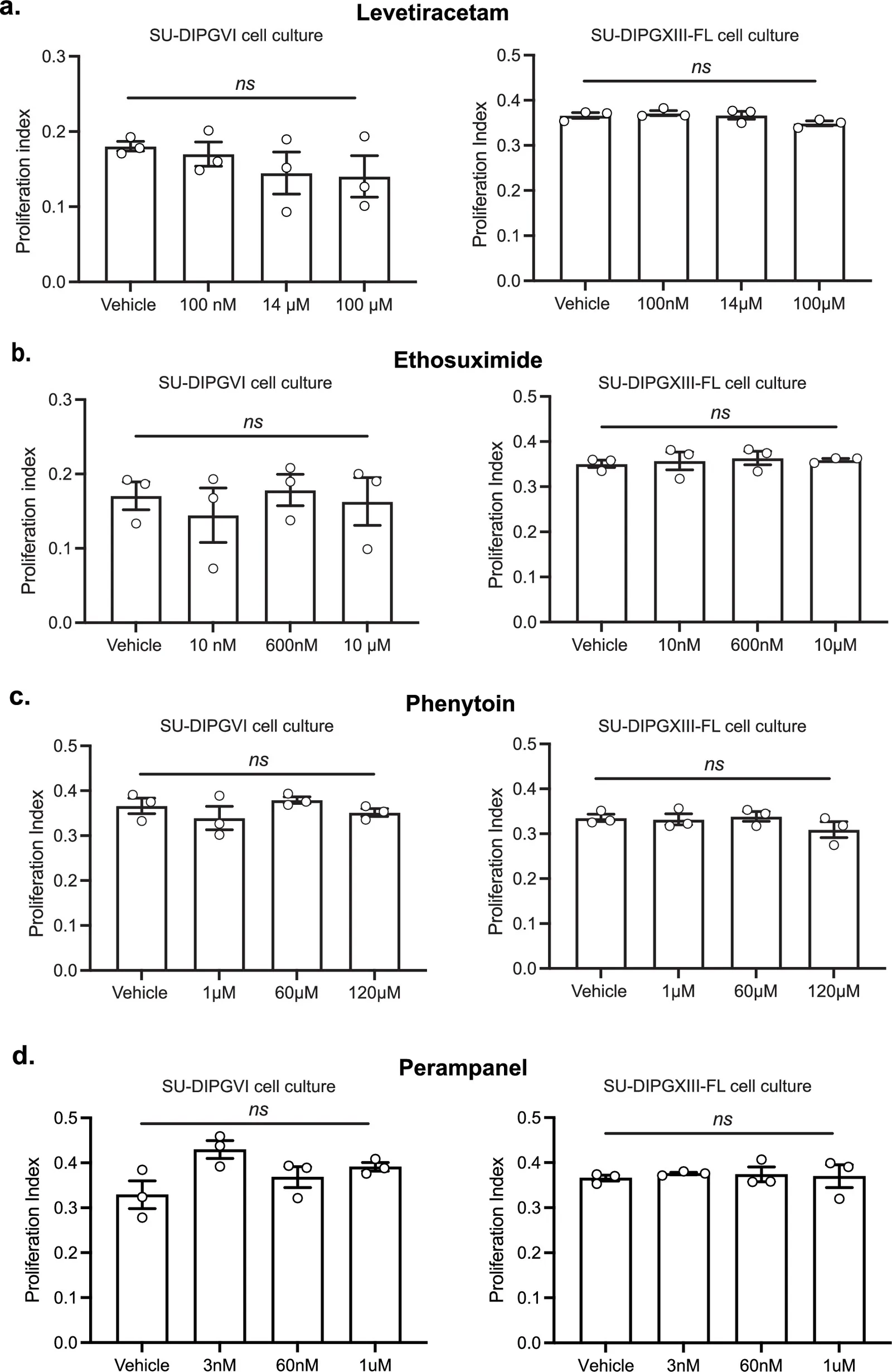
No effect of anti-seizure medications on diffuse midline glioma proliferation in monoculture. **a**. Levetiracetam treatment in patient-derived DMG cultures SU-DIPGVI and SU-DIPGXIII-FL had no effect on proliferation (n = 3 wells per group). One-way ANOVA. **b**. Ethosuximide treatment in patient-derived DMG cultures SU-DIPGVI and SU-DIPGXIII-FL had no effect on proliferation (n = 3 wells per group). One-way ANOVA. **c**. Phenytoin treatment in patient-derived DMG cultures SU-DIPGVI and SU-DIPGXIII-FL had no effect on proliferation (n = 3 wells per group). One-way ANOVA. **d**. Perampanel treatment in patient-derived DMG cultures SU-DIPGVI and SU-DIPGXIII-FL had no effect on proliferation (n = 3 wells per group). One-way ANOVA. All data are mean ± s.e.m. Source data

We next tested the survival benefit of levetiracetam treatment observed in retrospective patient data using preclinical mouse models. Using levetiracetam dosing to recapitulate clinically relevant regimens, we administered levetiracetam (20 mg/kg, 5 days a week for 4 weeks) to mice bearing patient-derived orthotopic H3K27M + DMG xenografts. Mice bearing patient-derived orthotopic DMG xenografts and treated with levetiracetam exhibited longer survival than vehicle-treated control mice (Fig. 3a,b). We observed reduced tumor burden over time in DMG-xenografted mice treated with levetiracetam (Fig. 3c), with a similar distribution of tumor spread (Fig. 3d,e). Concordantly, levetiracetam treatment decreased malignant glioma cell proliferation in mice bearing H3K27M + DMG xenografts compared to vehicle-treated controls, an effect observed in three independent patient-derived orthotopic DMG xenograft models and a genetically engineered murine allograft model (MADR^24^) of H3K27M + DMG (Figs. 3f–h and 4c). The effect of levetiracetam on glioma proliferation is dependent on interactions with the brain microenvironment rather than cell-intrinsic effects, as no effect of levetiracetam on DMG cell proliferation was observed in H3K27M + DMG monocultures (Extended Data Fig. 1a). In contrast to the antiproliferative effect of levetiracetam on H3K27M + DMG patient-derived xenografts and murine allografts, levetiracetam treatment did not significantly affect malignant glioma cell proliferation in mice bearing patient-derived IDH/H3 WT hemispheric HGG xenografts in three independent models of pediatric and adult H3/IDH WT hemispheric glioblastoma (Fig. 3i–k).

To determine whether the effects of levetiracetam on DMG proliferation and growth are generalizable to other antiseizure medications or more specific to levetiracetam, we tested two additional antiseizure medications with well-defined mechanisms of action: the T-type voltage-gated Ca^2+^ channel inhibitor ethosuximide and the voltage-gated Na^+^ inhibitor phenytoin. As expected, neither treatment impacted DMG growth in monoculture (Extended Data Fig. 1b,c). We also found no change in DMG cell proliferation in xenografted mice treated with ethosuximide or phenytoin compared to mice treated with vehicle control (Fig. 4a,b). This suggests that reducing neuronal excitability alone is not sufficient to reduce glioma proliferation.

Perampanel, an antiseizure medication that blocks AMPA receptor signaling, has been previously shown to reduce DMG growth through effects on glutamatergic neuron-to-glioma synapses^8^. Similar to levetiracetam, perampanel treatment had no effect on growth of DMG cells in monoculture (Extended Data Fig. 1d). To compare the effects of perampanel and levetiracetam on glioma growth, we treated mice bearing patient-derived orthotopic xenografts with levetiracetam, perampanel or vehicle control and found similar reductions in glioma proliferation in each treatment group compared to vehicle control (Fig. 4c). Interestingly, while both drugs increased mouse survival, levetiracetam did so to a greater extent than perampanel (Fig. 4d). A commonality between these medications that the other antiseizure drugs tested here do not share is that both perampanel and levetiracetam are known to directly target synaptic transmission. As the antiproliferative effect of levetiracetam is present in DMGs but not the hemispheric HGGs examined here, we hypothesized that levetiracetam may be specifically targeting neuron-to-glioma synaptic interactions that are enriched in DMGs compared to H3/IDH WT hemispheric HGGs.

### Levetiracetam targets GABAergic neuron-to-glioma synaptic transmission in DMGs

**Fig. 5:**
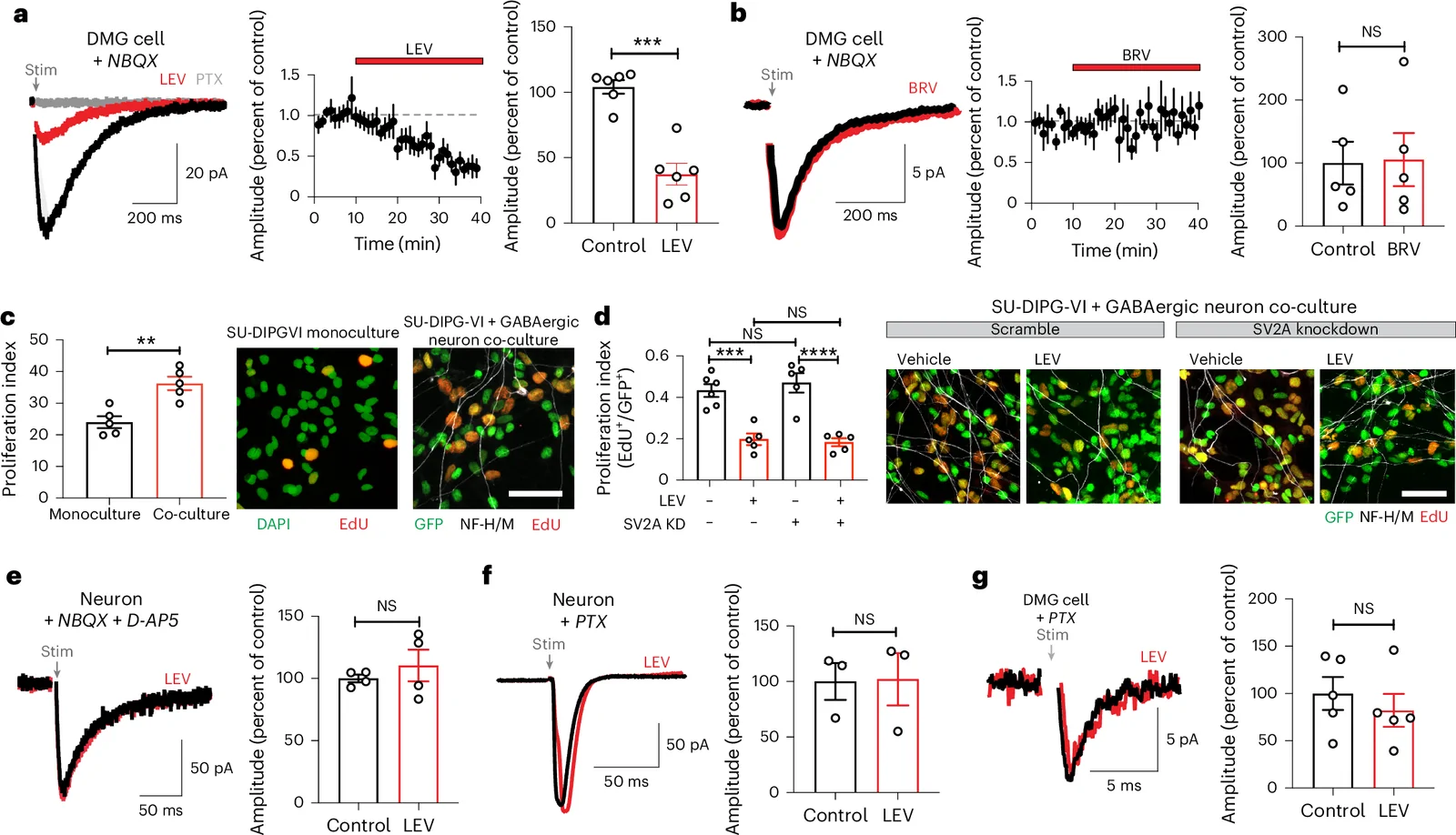
LEV selectively reduces GABAergic neuron-to-glioma synaptic currents. **a**, Left: representative averaged trace of GABAergic postsynaptic currents (PSCs) in DMG evoked by neuronal stimulation (Stim) before and after 30-min perfusion of 100 μM levetiracetam (LEV) and 50 μM picrotoxin (PTX), recorded in the presence of NBQX to block AMPA receptor-mediated currents. Center: time course of GABAergic PSC amplitude attenuation in response to LEV. Right: quantification of GABAergic PSC amplitude in DMG before and after 30-min LEV perfusion as a percentage of control (*n* = 6 cells from five mice, *P* = 0.0006). Two-tailed paired Student’s *t*-test. **b**, Left: representative averaged trace of GABAergic PSCs in DMG before and after 30-min perfusion of 30 μM brivaracetam (BRV), recorded in the presence of NBQX. Center: time course of GABAergic PSC amplitude in response to BRV. Right: quantification of GABAergic PSC amplitude in DMG before and after 30-min BRV perfusion as a percentage of control (*n* = 5 cells from three mice). Two-tailed paired Student’s *t*-test. **c**, A quantification of DMG cell (SU-DIPG-VI) proliferation (EdU^+^/GFP^+^) in monocultures or co-culture with GABAergic neurons (*n* = 5 wells per group, *P* = 0.0026). Unpaired, two-tailed Welch’s *t*-test. Right: confocal micrographs showing proliferating DMG cells (SU-DIPG-VI) in monoculture or in co-culture with GABAergic neurons. EdU: red, NF-H/M: white, DAPI: green (left image), GFP: green (right image). Scale bar, 50 µm. **d**, A quantification of DMG cell proliferation (EdU^+^/GFP^+^) in GABAergic neuron co-cultures transduced with scrambled or SV2A-targeting shRNA (SV2A KD) and treated with vehicle or LEV (*n* = 6 wells for scrambled/vehicle control, *n* = 5 for all other groups; *P* = 0.0004 for vehicle versus LEV in scramble control; *P* < 0.0001 for vehicle versus LEV in SV2A KD). One-way ANOVA with Tukey’s post hoc test. Right: confocal micrographs showing proliferation (EdU, red) of DMG cells expressing GFP (green) co-cultured with GABAergic neurons expressing NF-H/M (white). Scale bar, 50 μm. **e**, Representative averaged trace of GABAergic inhibitory PSC (IPSC) in hippocampal CA1 pyramidal neurons before and after 30-min perfusion of 100 μM LEV, recorded in the presence of NBQX and D-AP5. Right: quantification of current amplitude before and after LEV perfusion as a percentage of control (*n* = 4 cells from three mice). Two-tailed paired Student’s *t*-test. **f**, Representative averaged trace of glutamatergic excitatory PSC (EPSC) in CA1 pyramidal neurons before and after 30-min perfusion of 100 μM LEV, recorded in the presence of PTX. Right: quantification of current amplitude before and after LEV perfusion as a percentage of control (*n* = 3 cells from three mice). Two-tailed paired Student’s *t*-test. **g**, Representative averaged trace of glutamatergic PSCs in DMG before and after 30-min perfusion of 100 μM LEV, recorded in the presence of PTX. Right: quantification of current amplitude before and after LEV perfusion as a percentage of control (*n* = 5 cells from three mice). Two-tailed paired Student’s *t*-test. All data are mean ± s.e.m. NS, not significant, **P < 0.01, ***P < 0.001, ****P < 0.0001. Source data

**Extended Data Fig. 2:**
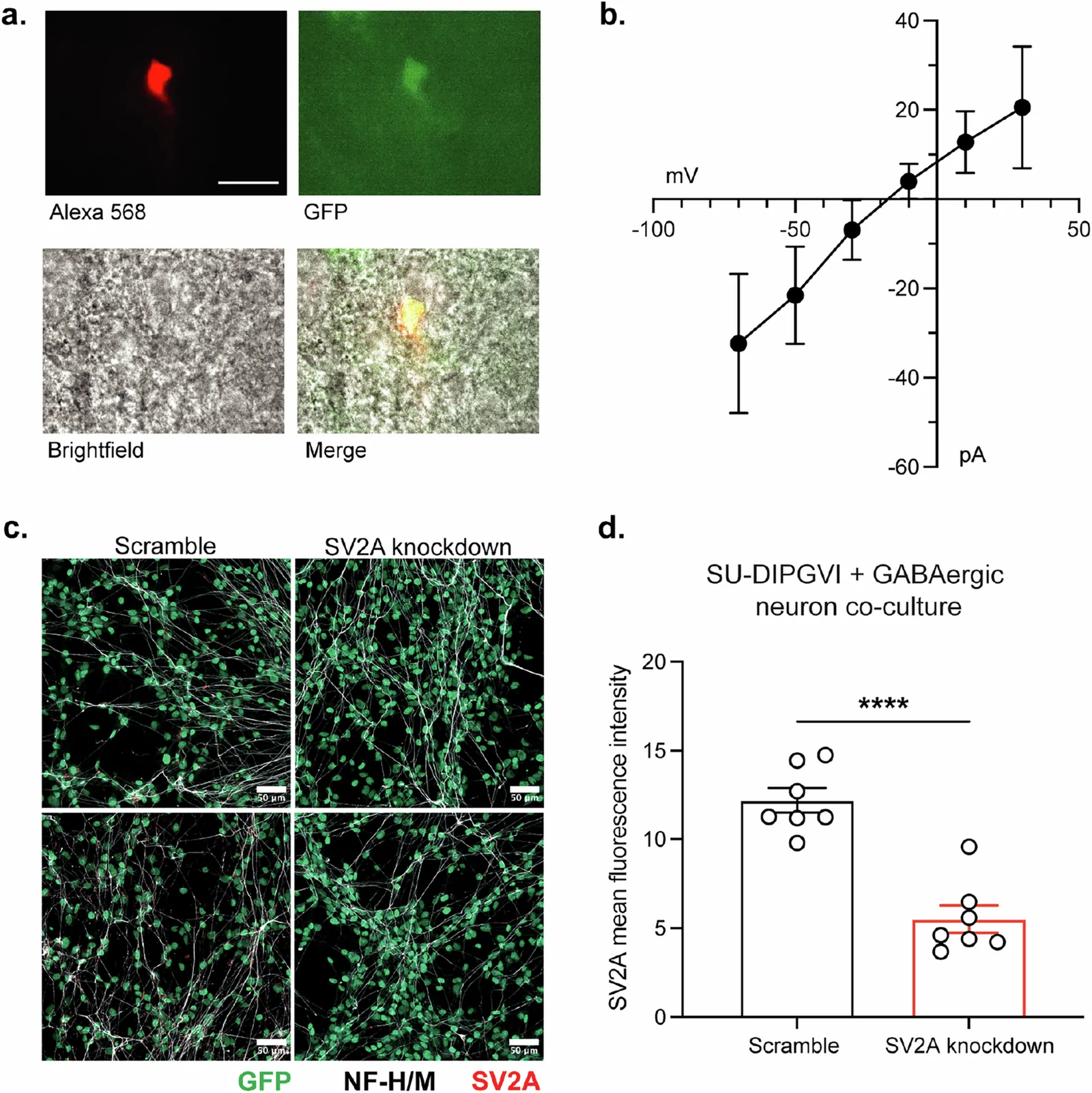
Confirmation of glioma patch and SV2A knockdown. **a**. Fluorescent and brightfield microscopy images showing a GFP+ glioma cell (green) dye-filled during patch-clamp recording (Alexa 568, red) and visible with brightfield imaging. Scale bar, 50 µm. n = 3 biological replicates. **b**. GABA current–voltage relationship of perforated patch recordings in LEV-treated DMG cells (SU-DIPG-VI; n = 4 cells from 3 mice). **c**. Confocal micrographs showing SV2A expression in GABAergic neuron-glioma co-cultures following scrambled shRNA (top) or SV2A knockdown (bottom) neuronal transduction. GFP: green, SV2A: red, NF-H/M: white. Scale bars, 50 µm. **d**. Quantification of mean fluorescence intensity of SV2A signal in GABAergic neurons transduced with scrambled or SV2A-targeting shRNA (n = 7 wells per group, p = <0.0001). Unpaired, two-tailed Welch’s t-test. All data are mean ± s.e.m. ****p < 0.0001. Source data

We recently discovered that depolarizing GABAergic currents mediated by neuron-to-glioma synapses drive malignant glioma proliferation in H3K27M-altered DMGs but not in H3/IDH WT hemispheric HGGs, which exhibit only minimal currents in response to GABA^11^. We thus wondered if levetiracetam may target these depolarizing GABAergic neuron-to-glioma synapses in DMG. To probe the effect of levetiracetam, we performed whole-cell patch-clamp electrophysiology of DMG cells (expressing GFP for visualization) in an acute slice preparation of xenografted mouse hippocampus^6,8,11^ (Extended Data Fig. 2a) and recorded GABAergic postsynaptic currents in response to low-frequency electrical stimulation of neurons in the microenvironment in the presence of glutamate receptor blockers. The recording electrodes contained a high intracellular Cl^‒^ concentration in order to clamp the postsynaptic level of *E*_GABA_ (the reversal potential of GABA-gated currents) to around 0 mV, thereby maintaining a constant driving force in the presence and absence of levetiracetam. Strikingly, application of levetiracetam led to an immediate, progressive decrease in the amplitude of the GABAergic postsynaptic currents in DMG cells (Fig. 5a). Previously^11^, we showed that the depolarizing action of GABA is based on active uptake of Cl^‒^ by the Na–K–Cl cotransporter, NKCC1^25^, which sets *E*_GABA_ at a very positive level (around −15 mV). Using perforated patch recordings to preserve the high native intracellular Cl^‒^ concentration in DMG cells, we recorded currents evoked by local GABA application and found that after levetiracetam exposure for >30 min, *E*_GABA_ was −17.6 ± 3.6 mV (Extended Data Fig. 2b). This value is similar to what we previously reported in untreated DMG cells^11^, indicating that levetiracetam does not affect the chloride concentration in glioma cells. Moreover, the *I*–*V* curves obtained in the presence of levetiracetam were similar to those obtained under control conditions^11^ suggesting that LEV is not acting to directly block the GABA_A_Rs in DMG cells. This is concordant with work on various neuronal cell types which have shown that levetiracetam does not directly inhibit GABA_A_Rs^26,27^.

A major mechanism of action for the antiseizure properties of levetiracetam is interaction with SV2A and consequent reduction of presynaptic vesicle release in hyperexcitable neurons^28–30^. To explore whether levetiracetam reduces DMG cell GABAergic currents through an SV2A-mediated mechanism, we tested brivaracetam, an antiseizure medication with an SV2A-specific mechanism of action. The potency of brivaracetam is high, such that it has a greater effect on high frequency postsynaptic currents at lower concentrations^31^ as compared to levetiracetam, for which the effect on high frequency postsynaptic currents in neurons is observed at 100 μM (refs. ^29,32^). Brivaracetam (30 μM) had no effect on DMG malignant cell GABAergic currents (Fig. 5b) using the same experimental paradigm in which these currents were reduced by levetiracetam. To further test the role of SV2A in the effect of levetiracetam on glioma proliferation, we knocked down SV2A in human induced pluripotent stem cells (iPS cell)-derived GABAergic neurons and co-cultured these neurons with DMG cells (Extended Data Fig. 2c,d). The presence of GABAergic neurons with normal levels of SV2A increased the proliferation of cultured glioma cells compared to monoculture (Fig. 5c). Treating the neuron-DMG co-culture with levetiracetam decreased glioma proliferation (Fig. 5d). shRNA-mediated knockdown of *SV2A* in GABAergic neurons achieved a ~55% reduction in SV2A protein expression (Extended Data Fig. 2d). Levetiracetam treatment reduced glioma proliferation similarly when DMG cells were co-cultured with GABAergic neurons treated with scramble control shRNA or with SV2A-targeting shRNA (Fig. 5d). Together, these data support the interpretation that the effect of levetiracetam on DMG proliferation and the GABAergic neuron-to-glioma synaptic response measured here is not mediated by an SV2A-dependent mechanism.

In the context of seizure-like experimental conditions, levetiracetam can reduce GABAergic synaptic currents in neurons, but this effect is contingent on high frequency stimulation and is not seen under non-seizure-like conditions^27^. We performed whole-cell patch-clamp recordings with a high-Cl^‒^ internal solution using the low-frequency stimulation experimental paradigm in which levetiracetam reduces GABAergic synaptic current amplitude in gliomas shown above and found no effect of levetiracetam on neuronal GABAergic synaptic currents (Fig. 5e). Thus, we conclude that there is an SV2A-independent mechanism, not dependent on high frequency stimulation, through which levetiracetam attenuates GABAergic currents in gliomas but not neurons. Under the same conditions (that is, low-frequency stimulation and 30-min incubation period), we found no effect of levetiracetam on neuron-to-neuron glutamatergic synaptic currents in the healthy neurons, as expected^29,33^ (Fig. 5f) nor on glutamatergic neuron-to-glioma synaptic currents (Fig. 5g). Taken together, these findings indicate a selectivity of levetiracetam action for neuron-to-glioma GABAergic synapses. The tumor-selective, SV2A-independent mechanism by which levetiracetam reduces DMG GABAergic synaptic currents and consequently tumor growth remains to be elucidated in future work.

## Discussion

Neuronal activity drives glioma proliferation through activity-dependent secretion of paracrine factors^2^ and through depolarization via direct neuron-to-glioma synapses^6,8,11^. Here, we target neuron–glioma interactions with the commonly used antiseizure drug levetiracetam and find that it reduces DMG malignant cell proliferation and extends survival in preclinical models of DMG but not of IDH/H3 WT hemispheric HGGs. Concordantly, retrospective, real-world patient data reveals that levetiracetam use correlates with extended survival in patients with DMG, but not those with non-DMG, hemispheric HGGs. Levetiracetam specifically attenuates GABAergic neuron-to-glioma synaptic currents through a tumor-selective, SV2A-independent mechanism. Future work will be required to determine this tumor-selective mechanism of levetiracetam action. GABA evokes depolarizing currents in DMG but not in IDH/H3 WT HGG^11^, which may explain the difference in the effects of levetiracetam on glioma malignant cell proliferation and patient survival between these glioma subtypes.

As shown here, antiseizure drugs appear to be growth-inhibitory only if the drug targets specific mechanisms of neuron–glioma interactions in that tumor type. Ethosuximide and phenytoin do not target known mechanisms of neuron–glioma interactions and did not affect tumor proliferation in the preclinical models used here. Similarly, levetiracetam did not influence the proliferation of the hemispheric (H3/IDH WT) HGG models used here. Past clinical studies of antiseizure drug effects in adult HGG have not been guided by knowledge of drugs that specifically target neurophysiological mechanisms operant in that tumor type. Not surprisingly, the results of such antiseizure medication studies have been mixed. Levetiracetam used concomitantly with chemoradiation has been reported to improve outcomes in hemispheric HGGs in some studies^18^, whereas large meta-analyses have found no discernable effect on outcome in others^19,20^. These discordant findings in the literature may reflect the heterogeneity inherent in hemispheric HGGs^20^, and specific subgroups of hemispheric HGGs yet to be determined could prove to be responsive to levetiracetam. Here, we found no effect of levetiracetam in three independent preclinical models of pediatric and adult hemispheric (H3/IDH WT) HGG and no effect of levetiracetam in retrospective analyses of pediatric patients with non-DMG, hemispheric HGGs.

Several limitations of this study are important to discuss. While the therapeutic potential of levetiracetam is supported by the retrospective clinical data and preclinical evidence presented here, it is important to note that prospective clinical trials are required to validate this conclusion. In this clinical retrospective study, it may be that those subjects who developed seizures and therefore received levetiracetam had tumors that are particularly neurotrophic and thus more susceptible to therapy with levetiracetam. It is also possible that tumors growing in neuroanatomical locations with relatively more GABAergic input or different GABA-dependent circuit dynamics are differentially affected by levetiracetam therapy. The brainstem, thalamus and spinal cord are rich in GABAergic inputs, and the importance of GABAergic signaling in brainstem DMG is underscored by the tumor growth-promoting effects of lorazepam—which positively modulates but does not directly stimulate GABA receptors—on pontine DMG growth^11^. An unknown confounder could also be associated with levetiracetam effects in retrospective patient cohorts. Future work, studying larger numbers of patients prospectively and stratifying subjects based on DMG location, molecular characteristics and glioma neuroscience correlative markers will be required to draw conclusions about the potential role of levetiracetam for DMG therapy.

Taken together, the findings here underscore the therapeutic potential of targeting powerfully growth-promoting neuron–glioma interactions with repurposed drugs that disrupt neurophysiological mechanisms hijacked by cancer.

## Methods

### Retrospective, real-world patient data

Retrospective data on patients with high-grade glial tumors were collected from patient databases at Stanford University (1990–2020) and the University of Michigan (2012–2021) through protocols approved by the respective institutional review boards (IRB). Database source data for pediatric patients with HGG were reviewed for this study to ensure veracity and completeness. For patients with tissue samples available (22 cases from Stanford University and University of Michigan), central review by a board-certified neuropathologist (H.V.) confirmed diagnoses. OS was calculated using the Kaplan–Meier estimator; the log-rank test was utilized to compare survival distributions. Patients were censored at time of last contact for the Kaplan–Meier analysis. Given the number of potential parameters with high correlation, an elastic net-regularized regression was utilized for covariate selection in a multivariable survival model. Clinical data including age, sex, tumor location, diagnosis of DMG and administration of ONC201, panobinostat, conventional chemotherapy and levetiracetam were considered potential covariates. A 20-fold cross-validation was used to obtain the value of *λ* that gave the minimum mean cross-validated error; corresponding coefficients for each variable were subsequently determined. All data were compiled and analyzed in R version 4.0 or higher. External validation cohorts for both entities, H3K27M DMG and pediatric hemispheric HGGs, were established from the University Medical Center Hamburg-Eppendorf (Germany), collected from patient records between 2006 and 2021. An additional validation cohort of patients with DMG was provided from the UCSF, collected from patient records between 2012 and 2022. Validation cohort data were analyzed using Prism v9.1.0 (GraphPad) software.

### Human samples and data

For all human tissue and cell studies, informed consent was obtained and tissue was used in accordance with protocols approved by the Stanford University IRB. IRB approval was also obtained for retrospective analyses of real-world clinical data kept in IRB-approved databases at Stanford University, University Michigan and UCSF. For the cohort from University Medical Center Hamburg-Eppendorf, informed written consent was obtained from all patients or their legal guardians, and further data processing was approved by the medical ethics committee of the Hamburg Chamber of Physicians (PV4904).

### Mice and housing conditions

All in vivo experiments were conducted in accordance with protocols approved by the Stanford University Institutional Animal Care and Use Committee (IACUC) and performed in accordance with institutional guidelines. Animals were housed according to standard guidelines at 21 °C with free access to food and water in a 12 h light–12 h dark cycle. For brain tumor xenograft experiments, the IACUC does not set a limit on maximal tumor volume but rather on indications of morbidity. In no experiments were these limits exceeded as mice were killed if they exhibited signs of neurological morbidity or if they lost 15% or more of their body weight.

### Orthotopic xenografting and allografting

For all patient-derived xenograft studies, NSG mice (NOD-SCID-IL2R gamma chain-deficient, The Jackson Laboratory) were used. For allograft studies, C57BL/6J mice (The Jackson Laboratory) were used. Male and female mice were used equally. A single-cell suspension from cultured SU-DIPG-VI, SU-DIPG-XIII-FL, SU-DIPG-50, SU-pcGBM2, SU-DIPG-36, SU-DIPG-XIII-P, SF0232 or SF0238 patient-derived glioma neurospheres^8,16^ or cultured cells derived from an electroporation-induced genetic mouse model of H3K27M DMGs (MADR^24,34^) were prepared in sterile phosphate-buffered saline (PBS) immediately before the xenograft or allograft procedure. All DMG models express GFP except SU-DIPG-XIII-P (pons). Animals at postnatal day (P)28–30 were anaesthetized with 1–4% isoflurane and placed in a stereotactic apparatus. The cranium was exposed via midline incision under aseptic conditions. Approximately 300,000 cells in 3 µl sterile PBS were stereotactically implanted through a 26G burr hole, using a digital pump at infusion rate of 0.4 µl min^−1^ and 26G Hamilton syringe. For all electrophysiology and optogenetics experiments, cells were implanted into the CA1 region of the hippocampus (1.5 mm lateral to midline, −1.8 mm posterior to bregma, −1.4 mm deep to cranial surface). SU-DIPG-XIII-FL, SU-pcGBM2, SF0232 and SF0238 for levetiracetam treatments were xenografted into the premotor cortex (0.5 mm lateral to midline, 1.0 mm anterior to bregma, −1.75 mm deep to cranial surface). SU-DIPG-XIII-P and SU-DIPG-36 for survival studies and SU-DIPG-VI, SU-DIPG-50 and MADR for levetiracetam, ethosuximide, phenytoin and perampanel treatments were xenografted or allografted into the pons (1.0 mm lateral to midline, −0.8 mm posterior to lambda, −5.0 mm deep to cranial surface). At the completion of infusion, the syringe needle was allowed to remain in place for a minimum of 2 min and then manually withdrawn at a rate of 0.875 mm min^−1^ to minimize backflow of the injected cell suspension.

### Patient-derived and mouse model-derived cell culture

HGG cultures SU-DIPG-VI, SU-DIPG-XIII-FL, SU-DIPG-50, SU-pcGBM2, SU-DIPG-36 and SU-DIPG-XIII-P were generated from patient tissue samples as previously described^2,3^. In brief, tissue was obtained from HGG (World Health Organization grade 3 or 4) tumors at the time of biopsy or from early post-mortem donations in accordance with IRB-approved protocols. Tissue was dissociated both mechanically and enzymatically and grown in a defined, serum-free medium designated ‘tumor stem media’, consisting of neurobasal(-A) (Invitrogen), B27(-A) (Invitrogen), human bFGF (20 ng ml^−1^, Shenandoah), human EGF (20 ng ml^−1^, Shenandoah), human PDGF-AA (10 ng ml^−1^) and PDGF-BB (10 ng ml^−1^, Shenandoah) and heparin (2 μg ml^−1^, Stem Cell Technologies). For all patient-derived cultures, mycoplasma testing was routinely performed, and short tandem repeat DNA fingerprinting was performed every 3 months to verify authenticity. The short tandem repeat fingerprints and clinical characteristics for the patient-derived cultures and xenograft models used have been previously reported^2,3^ Patient-derived HGG cells SF0232 and SF0238 were a generous gift from Shawn Hervey-Jumper at UCSF. For the murine MADR DMG model cells, culture was performed similarly as previously described^24,34^.

### Mouse drug treatment studies

For histological analysis of tumor proliferation and tumor burden in patient-derived xenografts, NSG mice were xenografted as above with SU-DIPG-VI, SU-DIPG-XIII-FL, SU-DIPG-50, SU-pcGBM2, SF0232 or SF0238 cells and randomized to treatment group by a blinded investigator. A total of 4–6 weeks postxenograft, mice were treated with systemic administration of levetiracetam (20 mg kg^−1^, Selleck Chemicals) or phenytoin (50 mg kg^−1^, Selleck Chemicals) via intraperitoneal injection or ethosuximide (300 mg kg^−1^, Selleck Chemicals) or perampanel (0.75 mg kg^−1^, Adooq Biosciences) via oral gavage for 4 weeks (once a day administration, 5 days per week).

For the MADR murine DMG model, C57BL/6J mice were allografted as above and randomized to treatment group by a blinded investigator. A total of 10 days after allografting, mice were treated with systemic administration of levetiracetam (20 mg kg^−1^, Selleck Chemicals) via intraperitoneal injection for 4 weeks (5 days per week).

For all studies, controls were treated with an identical volume of the relevant vehicle. Bioluminescence imaging was performed before treatment and every 7 days thereafter using an IVIS imaging system (Xenogen) under isoflurane anesthesia.

For survival studies, SU-DIPG-36 or SU-DIPG-XIII-P cells were xenografted to pons as above, and levetiracetam was administered (20 mg kg^−1^ intraperitoneal injection), beginning 3 days after xenografting and administered 5 days a week for 28 days. Morbidity criteria used were either reduction of weight by 15% initial weight or severe neurological motor deficits consistent with brainstem dysfunction (that is, hemiplegia or an incessant stereotyped circling behavior seen with ventral midbrain dysfunction). Kaplan–Meier survival analysis using log-rank testing was performed to determine statistical significance.

### Perfusion and immunohistochemistry

Animals were anaesthetized with intraperitoneal avertin (tribromoethanol) and then transcardially perfused with 20 ml of PBS. Brains were fixed in 4% PFA overnight at 4 °C and then transferred to 30% sucrose for cryoprotection. Brains were then embedded in Tissue-Tek OCT (Sakura) and sectioned in the coronal plane at 40 µm using a sliding microtome (Microm HM450, Thermo Scientific).

For immunohistochemistry, coronal sections were incubated in blocking solution (3% normal donkey serum, 0.3% Triton X-100 in TBS) at room temperature for 1–2 h. Chicken anti-GFP (1:500, Abcam), mouse antihuman nuclei clone 235-1 (1:100, Millipore) and rabbit anti-Ki67 (1:500, Abcam) were diluted in antibody diluent solution (1% normal donkey serum in 0.3% Triton X-100 in TBS) and incubated 24–36 h at 4 °C. Sections were then rinsed three times in TBS and incubated in secondary antibody solution containing Alexa 488 donkey antichicken IgG, Alexa 594 donkey antimouse IgG, Alexa 594 donkey antirabbit IgG, Alexa 647 donkey antimouse IgG or Alexa 647 donkey antirabbit IgG, used at 1:500 (Jackson ImmunoResearch) in antibody diluent at 4 °C for 2 h. Sections were rinsed three times in TBS and mounted with ProLong Gold Mounting medium (Life Technologies).

### Confocal imaging

Images were acquired using a 40× oil immersion objective or 20× air objective of a Zeiss LSM700, Zeiss LSM800 or Zeiss LSM980 scanning confocal microscope and Zen imaging software (Carl Zeiss). Cell quantification within xenografts was performed by a blinded investigator.

### Quantification of cell proliferation, tumor burden and tumor spread

For Ki67 analysis, three fields for quantification were selected from each of three consecutive sections in a 1-in-6 series of 40-μm coronal sections with respect to overall tumor burden. Within each field, all HNA-positive and GFP-positive tumor cells were quantified to determine tumor burden within the areas quantified. HNA-positive cells were then assessed for co-labeling with Ki67. To calculate the proliferation index (the percentage of proliferating tumor cells for each mouse), the total number of HNA-positive cells co-labeled with Ki67 across all areas quantified was divided by the total number of cells counted across all areas quantified (Ki67^+^/HNA^+^). For analysis of tumor burden and tumor spread, all slices containing tumor cells were imaged from a 1-in-2 series of 40-μm coronal sections. Images of whole brain slices were adjusted for threshold and analyzed in ImageJ. In brief, a polygon section containing the tumor cells was traced and measured for area (for tumor spread) and mean intensity (for tumor burden).

### EdU incorporation assay

DIPG tumor neurosphere cultures SU-DIPG-VI, SU-DIPG-XIII and SU-DIPG-50 were generated as previously described^2,3^ from early post-mortem tissue donations and grown as tumor neurospheres in defined, serum-free ‘tumor stem medium’, consisting of 1:1 mixture of neurobasal(-A) (Invitrogen) and D-MEM/F-12 (Invitrogen), HEPES buffer (Invitrogen), MEM sodium pyruvate (Invitrogen), MEM non-essential amino acids (Invitrogen), GlutaMAX-1 supplement (Invitrogen), B27(-A) (Invitrogen), human bFGF (20 ng ml^−1^, Shenandoah), human EGF (20 ng ml^−1^, Shenandoah), human PDGF-AA (10 ng ml^−1^) and PDGF-BB (10 ng ml^−1^, Shenandoah) and (2 μg ml^−1^, Stem Cell Technologies).

A total of 100,000 glioma cells were plated onto circular glass coverslips (Electron Microscopy Services) pretreated for 1 h at 37 °C with poly-ʟ-lysine (Sigma) and then 1 h at 37 °C with 10 µg ml^−1^ natural mouse laminin (Thermo Fisher). Dimethyl sulfoxide (Sigma-Aldrich) or drugs at the concentrations indicated (dissolved in dimethyl sulfoxide) were added to the coverslips. A total of 10 μM EdU was added to each coverslip. Cells were fixed after 24 h using 4% paraformaldehyde in PBS and stained using the Click-iT EdU kit and protocol (Invitrogen). Proliferation index was then determined by quantifying the fraction of EdU labeled cells/DAPI-labeled cells using confocal microscopy.

### Slice preparation for electrophysiology

Coronal slices (300-µm thick) containing the hippocampal region were prepared from mice (at least 8 weeks after xenografting) in accordance with a protocol approved by Stanford University IACUC. After rapid decapitation, the brain was removed from the skull and immersed in ice-cold slicing artificial cerebrospinal fluid (ACSF) containing: 125 mM NaCl, 2.5 mM KCl, 25 mM glucose, 25 mM NaHCO_3_ and 1.25 mM NaH_2_PO_4_, 3 mM MgCl_2_ and 0.1 mM CaCl_2_. After cutting, slices were incubated for 30 min in warm (30 °C) oxygenated (95% O_2_, 5% CO_2_) recovery ACSF containing: 100 mM NaCl, 2.5 mM KCl, 25 mM glucose, 25 mM NaHCO_3_, 1.25 mM NaH_2_PO4, 30 mM sucrose, 2 mM MgCl_2_ and 1 mM CaCl_2_ before being allowed to equilibrate at room temperature for an additional 30 min.

### Electrophysiology

Slices were transferred to a recording chamber and perfused with oxygenated, warmed (28–30 °C) recording ACSF containing: 125 mM NaCl, 2.5 mM KCl, 25 mM glucose, 25 mM NaHCO_3_, 1.25 mM NaH_2_PO_4_, 1 mM MgCl_2_ and 2 mM CaCl_2_. Cells were visualized using a microscope equipped with DIC optics (Olympus BX51WI). For glioma cell recordings and neuronal inhibitory postsynaptic current recordings, patch pipettes were filled with CsCl-based pipette solution containing: 150 mM CsCl, 5 mM EGTA, 1 mM MgCl_2_, 10 mM HEPES, 2 mM ATP, 0.3 mM GTP, pH 7.3. For neuronal excitatory postsynaptic currents recordings, patch pipettes were filled with K-gluconate-based pipette solution containing: 20 mM KCl, 100 mM K-gluconate, 10 mM HEPES, 4 mM Mg-ATP, 0.3 mM Na-GTP, 10 mM Na-phosphocreatine, 0.2 mM EGTA, pH 7.3. Patch electrodes had resistances of 4–5 MΩ. Pipette solution additionally contained Alexa 568 (50 μM) to visualize the cell through dye-filling during whole-cell recordings. Gramicidin A (60 μg ml^−1^) was added to the pipette solution for perforated patch recordings. A perforated patch was considered satisfactory when an access resistance of ~30 MΩ was obtained about 30 min after attaining gigaseal resistance. Leak of Alexa 568 dye from the pipette into the cell during perforated patch recordings indicates a damaged membrane, and the data from such recordings were discarded. *E*_GABA_ was calculated for each individual cell and averaged. Glioma cells were voltage-clamped at a holding potential of −70 mV. For all experiments, series resistance was <30 MΩ. Synaptic responses in gliomas and neurons were evoked with a bipolar electrode connected to an Iso-flex stimulus isolator (AMPI) placed 100–200 μm from the patched cells to stimulate local interneurons or Schaffer collaterals. A low intensity stimulation, sufficient to evoke consistent responses but not higher, was used at a frequency of 0.1 Hz throughout the experiment, including 10 min of baseline recordings and 30 min of levetiracetam or brivaracetam perfusion. For *E*_GABA_ recordings, GABA (1 mM) in recording ACSF was applied via a puff pipette, which was placed approximately 100 μm away from the patched cell and controlled by a Picospritzer II (Parker Hannifin Corp.), and tetrodotoxin (0.5 µM) was perfused with the recording ACSF to prevent neuronal action potential firing. Signals were acquired with a MultiClamp 700B amplifier (Molecular Devices) and digitized at 10 kHz with an Axon Digidata 1550B (Molecular Devices) or an InstruTECH LIH 8 + 8 data acquisition device (HEKA). Data were recorded and analyzed using pClamp 11 software suite (Molecular Devices), AxoGraph X (AxoGraph Scientific) and/or IGOR Pro 8 (Wavemetrics). For representative traces, stimulus artifacts preceding the synaptic responses have been removed for clarity.

### Pharmacological agents

Drugs and toxins used for electrophysiology were picrotoxin (50 µM, Tocris), NBQX (10 µM, Tocris), D-AP5 (100 µM, Tocris), tetrodotoxin (0.5 µM, Tocris), levetiracetam (100 μM, Selleck Chemicals) and brivaracetam (30 μM, UCB). When used for in vitro slice application, drugs were made up as a stock in distilled water or dimethyl sulfoxide and dissolved to their final concentrations in ACSF before exposure to slices. Final concentration of dimethyl sulfoxide was <1%.

### SV2A knockdown in human iPS cell-derived GABAergic neurons

Human iPS cell-derived GABAergic neurons (Bit.bio io1003) were used for neuron–glioma co-culture assays. 96-well µ-plates (ibidi 89607) were coated overnight at room temperature with 0.01% poly-L-ornithine (Sigma-Aldrich P4957), washed 3× with sterile water and subsequently coated for 2 h at 37 °C with laminin (R&D Systems 3400-010-02) diluted in sterile PBS. Cells were seeded immediately after laminin removal at 3 × 10^4^ cells per well in 100 µl comp:GS medium (DMEM/F-12, N2 (1×), MEM NEAA (1×) and doxycycline (1 µg ml^−1^)). Cryovials were thawed at 37 °C, diluted dropwise with DMEM/F-12 (Thermo Fisher 11330032) and centrifuged at 200*g* for 5 min at room temperature. Cell pellets were resuspended in comp:GS + R stabilization medium (comp:GS supplemented with 10 µM ROCK inhibitor Y-27632; Abcam Ab144494) and cultured for 72 h. At 24 h post thaw (day 3), 90% of the medium was replaced with comp:GS. After 72 h, media were switched to maintenance medium (comp:MM: Neurobasal supplemented with B27 (1×), GlutaMAX (1×) and BDNF (10 ng ml^−1^)). Half-media changes were performed every 48 h.

For SV2A knockdown, neurons were transduced 72 h after thawing using human shRNA lentiviral particles (OriGene TL308996V) or scrambled control. Viral particles were mixed with lentiviral transduction enhancer (System Biosciences LV860A-1) according to the manufacturer’s instructions and applied to cells for 48 h, followed by a full medium replacement with comp:MM. Efficient SV2A knockdown was validated by immunofluorescence.

A total of 14 days after cell induction, glioma cells (SU-DIPG-VI-GFP, 1.5 × 10^4^ cells per well) were added to neuronal cultures and co-incubated for 48 h in comp:MM. Subsequently, 4 µM 5-ethynyl-2′-deoxyuridine (EdU) was added with or without levetiracetam and incubated for an additional 24 h. Cultures were fixed with 4% paraformaldehyde for 20 min at room temperature. EdU staining was performed using the manufacturer’s protocol (Click-iT EdU Imaging Kit, Thermo Fisher C10339). Cells were stained overnight at 4 °C with chicken antineurofilament-H/M (1:500, Antibodies Incorporated AB_2313554) and rabbit anti-SV2A (1:500, Synaptic Systems 119 003). After washing, cells were incubated for 2 h at room temperature with species-matched Alexa Fluor secondary antibodies (1:500, Jackson ImmunoResearch 703-605-155 and 711-585-152). Wells were mounted with antifade medium (ProLong Gold, Thermo Fisher P36930) and imaged using a Zeiss LSM980 confocal microscope. SV2A knockdown was quantified by mean fluorescence intensity of SV2A staining. Proliferation index was determined by quantifying the fraction of EdU labeled cells/GFP labeled cells.

### Statistical analyses

Statistical tests were conducted using Prism v9.1.0 (GraphPad) software. Gaussian distribution was confirmed by the Shapiro–Wilk normality test. For parametric data, unpaired two-tailed Student’s *t*-test or one-way analysis of variance (ANOVA) with Tukey’s or Dunnett’s post hoc test to examine pairwise differences and/or test for linear contrast were used as indicated in figure legends. Paired two-tailed Student’s *t*-tests or repeated measures one-way ANOVA with Dunnett’s post hoc analysis were used in electrophysiological experiments within the same cell. Simple linear regression analysis was used to determine the *x* intercept in current–voltage relationship experiments. Two-tailed log-rank analyses or Gehan–Breslow–Wilcoxon analyses were used to analyze statistical significance of Kaplan–Meier survival curves. Statistical test results are reported in Figs. 1–5 and Extended Data Figs. 1 and 2. At least three mice for in vivo experiments, and at least three independent coverslips for in vitro experiments, were used per test group to attain 80% power to detect an effect size of 20% at significance level 0.05. Statistical analyses of retrospective patient data are described above.

### Reporting summary

Further information on research design is available in the Nature Portfolio Reporting Summary linked to this article.

## Online content

Any methods, additional references, Nature Portfolio reporting summaries, source data, extended data, supplementary information, acknowledgements, peer review information; details of author contributions and competing interests; and statements of data and code availability are available at https://doi.org/10.1038/s41591-026-04646-6.

## Supplementary information

Supplementary Information Supplementary Tables 1 and 2 and source data files.

Reporting Summary

Peer Review File

## Source data

Source data Fig. 1 Statistical source data.

Source data Fig. 2 Statistical source data.

Source data Fig. 3 Statistical source data.

Source data Fig. 4 Statistical source data.

Source data Fig. 5 Statistical source data.

Extended Data Fig. 1 Statistical source data.

Extended Data Fig. 2 Statistical source data.

## Data Availability

Data are available within Supplementary Information. Source data are provided with this paper.
