# Supplementary material for: Levetiracetam therapeutically targets GABAergic synapses in diffuse midline glioma
Source: Nat Med. 2026 Sep 17;32(10):3792–800. doi: 10.1038/s41591-026-04646-6 (PMC13668697; doi:10.1038/s41591-026-04646-6)
Supplement: Supplementary file 1 [file 41591_2026_4646_MOESM1_ESM.pdf]

nature medicine

Supplementary information

https://doi.org/10.1038/s41591-026-04646-6

Levetiracetam therapeutically targets GABAergic synapses in diffuse midline glioma

In the format provided by the authors and unedited

Supplementary Table 1.

Institution	Age (years)	Sex	Diagnosis	Other treatments	Levetiracetam yes/no	Overall survival (days)
University of Michigan	7	Male	Pontine diffuse midline glioma, H3.3K27M, ATRX Q119*, PPM1D G463fs, PDGFRA amplification, KIT, KDR	XRT	Yes	638
University of Michigan	12	Male	Thalamic diffuse midline glioma, H3.3K27M, TP53 H179N, ATRX R2197C, TRIO I780fs deletion, subclonal WAS exon 8 loss, Focal amplifications: HGF, MET, GAB2, TBCD Copy gain NTRK2 (4 copies)	XRT, TMZ, lomustine, Cabozantinib, subtotal resection	Yes	362
University of Michigan	9	Female	Thalamic diffuse midline glioma, H3.1K27M, EGFR A289V, MAX R60Q, PIK3CA R88Q, ARHGAP35 G854E, Subclonal: CEBPZ D78N, DIS3 I779F, MSH3 P64A, BCORL1 K1421fs deletion; subclonal E2F7 L6fs deletion; Copy gain: Chr1q, 2, 5, 7, 15p; UPD: Chr12	XRT, TMZ, Vimpat, ONC201	Yes	659
University of Michigan	13	Female	Thalamic diffuse midline glioma H3K27M, BRAF V600E	XRT, TMZ, Dabrafenib, trametinib, Bevacuzimab, Irinotecan, ONC201, subtotal resection	Yes	Censored at 745
University of Michigan	8	Male	Thalamic diffuse midline glioma, BRAF V600E, H3WT	XRT, Dabrafenib, trametinib, Trileptal, gabapentin, Vimpat	Yes	Censored at 540
University of Michigan	13	Male	Thalamic diffuse midline glioma	XRT, TMZ, ONC201, Oxcarbazine, Zonisamide	Yes	617
University of Michigan	20	Male	Pontine diffuse midline glioma, H3K27M mutant	XRT, TMZ, Depakote, ONC201	Yes	Censored at 446
University of Michigan	10	Female	Thalamic diffuse midline, FBXW R465H, STAG2 R110, SETD2 R2165, VAV1 657A, FGFR3 copy gain, CDK11A/B homozygous deletion, gene fusion FGFR3-PHGDH, FGFR3 copy gain, Gain: chromosomes 1q and 7q; Loss: chromosomes 1p, 4p15-16, 6q, and 10q, H3WT	XRT, COG clinical trial ACNS0831, ponatinib	Yes	Censored at 1249

University of Michigan	10	Male	Thalamic diffuse midline glioma, H3K27M mutation; two TP53 mutations, IDH-1 negative, no MGMT methylation	XRT, ONC201, Bevacizumab, Vimpat, subtotal resection	Yes	Censored at 542
University of Michigan	12	Male	Thalamic diffuse midline glioma, H3K27M mutation; ATRX pR1803H, recurrent (ChrX), NTRK2 internal tandem duplication, focal amplification	XRT, TMZ, ONC201, bevacizumab, entrectinib, subtotal resection	Yes	Censored at 454
Stanford University	6	Female	Pontine diffuse midline glioma	XRT, Etoposide	Yes	407
Stanford University	2	Female	Pontine diffuse midline glioma	XRT	Yes	503
Stanford University	11	Female	Thalamic diffuse midline glioma, H3WT, IDH1/2 WT, ATRX and TP53 mutated	XRT, TMZ, Lomustine, Savolitinib	Yes	874
Stanford University	13	Female	Thalamic diffuse midline glioma, H3K27-altered	No XRT, alternative therapies only	Yes	56
Stanford University	11	Male	Thalamic diffuse midline glioma	XRT, TMZ, etoposide	Yes	436
University of Michigan	17	Male	Pontine/spinal diffuse midline glioma, H3.3 K27M, TP53 H179R, ATRX D1313fs, ERCC5 K917fs, Homozygous loss: NF1 Copy loss: BRCA1, CDK12	XRT, SAHA (HDAC inhibitor), subtotal resection	No	316
University of Michigan	5	Female	Pontine diffuse midline glioma, PIK3CA; H3.1 K27M	XRT, everolimus, bevacuzimab, panobinostat, subtotal resection	No	Censored at 588
University of Michigan	13	Male	Pontine diffuse midline glioma, H3.3K27M mutation; FGFR3 activating mutation, loss of BCOR	XRT, bevacizumab, ponatinib, Panobinostat, pazopanib, everolimus subtotal resection	No	Censored at 533
University of Michigan	12	Male	Thalamic diffuse midline glioma, somatic mutations in EGFR V292L, mutation/loss in CDKN2C and BCOR1, EGFR T483_G485del, gene fusion ELF4-SMARCA1, H3WT	Proton XRT, TPCV, Osimertinib, bevacizumab	No	Censored 502
University of Michigan	8	Male	Pontine diffuse midline glioma, H3.3K27M	XRT, COG phase 1 trial (ADVL1217), everolimus, panobinostat, Depakote	No	Censored at 259
University of Michigan	2	Male	Pontine diffuse midline glioma, H3.3K27M, TP53 R273C, CTTNBP2-MET, in frame with MET kinase domain	XRT, cabozantinib	No	Censored at 246

University of Michigan	9	Female	Pontine diffuse midline glioma, H3.3K27M, PPM1D E525, TP53 R248W, additional non-recurrent missense mutations: AFF2 T385N, ABCC1 T826M, PIK3C2G S1183R, PTPRJ E841fs; Copy gain: Chr 1q, 8q, 17q; Loss of Chr11q	Proton XRT, XRT, ONC201	No	
University of Michigan	1	Female	Pontine/thalamic diffuse midline glioma, H3K27M	NA	No	Censored at 29
University of Michigan	7	Female	Pontine diffuse midline glioma, H3.1 K27M, ACVR1 R206H, USP9X splice acceptor, exon33, BCOR A603fs deletion	XRT, ONC201 (arm a), bevacizumab	No	Censored at 626
University of Michigan	13	Male	Thoracic spine diffuse midline glioma, H3.3 K27M, ATRX mutation, FGFR1, H3.3 G34W, PPM1D, PTPN11	XRT, ONC201, bevacizumab, subtotal resection	No	Censored at 398
University of Michigan	17	Female	Thalamic diffuse midline glioma, H3.3, ATRX, PI3KR1, TP53 mutations	Proton XRT, PTC56, ONC201, Subtotal resection	No	Censored at 216
University of Michigan	2	Female	Thalamic diffuse midline glioma, H3K27M	Proton XRT, ONC201	No	Censored at 176
University of Michigan	9	Female	Pontine diffuse midline glioma, H3.3K27M, PDGFA A153T, TP53 P222_E224delinL, IGF1R focal amplification	Proton XRT, ONC201, bevacizumab	No	Censored at 459
University of Michigan	9	Male	Tectal/thalamic diffuse midline glioma, H3K27M mutation, KRAS 212R mutation, HGF amplification	Proton XRT, vincristine, carboplatin, trametinib, ONC201, Subtotal resection	No	Censored at 2220
Stanford University	6	Female	Pontine diffuse midline glioma	XRT, Cisplatin	No	182
Stanford University	16	Male	Pontine diffuse midline glioma	XRT	No	7
Stanford University	5	Male	Pontine diffuse midline glioma	XRT, TMZ, Vorinostat	No	424
Stanford University	7	Male	Pontine diffuse midline glioma	XRT, TMZ	No	50
Stanford University	7	Female	Pontine diffuse midline glioma	XRT, TMZ, Valproic Acid	No	416
Stanford University	15	Male	Pontine diffuse midline glioma	XRT	No	125
Stanford University	9	Male	Pontine diffuse midline glioma	XRT	No	180

Stanford University	13	Male	Pontine diffuse midline glioma, H3.1K27M, MAPKAPK2 gain	XRT, Arsenic Trioxide	No	625
Stanford University	5	Male	Pontine diffuse midline glioma	XRT	No	58
Stanford University	7	Female	Thalamic diffuse midline glioma	XRT, TMZ, Arsenic Trioxide, Bevacizumab, Carmustine, Eroltinib, Irinotecan, Sirolimus, diazepam, phenobarbital, Subtotal Resection	No	1055
Stanford University	9	Female	Pontine diffuse midline glioma	XRT, TMZ	No	231
Stanford University	5	Female	Pontine diffuse midline glioma	XRT	No	110
Stanford University	4	Female	Pontine diffuse midline glioma	XRT, nivolumab	No	Censored at 8
Stanford University	10	Female	Thalamic diffuse midline glioma, H3K27-altered	XRT, TMZ	No	Censored at 1241
Stanford University	4	Female	Pontine diffuse midline glioma	NA	No	116
Stanford University	3	Male	Pontine diffuse midline glioma	NA	No	437
Stanford University	9	Male	Medulla diffuse midline glioma	XRT, TMZ, Bevacizumab, Imetelstat, Carmustine, Subtotal Resection	No	2938
Stanford University	3	Female	Pontine diffuse midline glioma	NA	No	35
Stanford University	8	Female	Pontine diffuse midline glioma	XRT	No	Censored at 53
Stanford University	8	Female	Pontine diffuse midline glioma	XRT	No	281
Stanford University	8	Male	Pontine diffuse midline glioma	XRT	No	197
Stanford University	17	Male	Ponto-medulla diffuse midline glioma	XRT, TMZ, Bevacizumab	No	2324
Stanford University	6	Female	Pontine diffuse midline glioma, H3.3K27M, HIST1H3B gain	XRT	No	54
Stanford University	6	Female	Pontine diffuse midline glioma	NA	No	13
Stanford University	4	Male	Pontine diffuse midline glioma	NA	No	141

Stanford University	6	Male	Pontine diffuse midline glioma	XRT	No	106
Stanford University	4	Male	Pontine diffuse midline glioma	XRT, Arsenic Trioxide	No	205
Stanford University	4	Male	Pontine diffuse midline glioma	XRT	No	397
Stanford University	7	Male	Pontine diffuse astrocytoma (WHO III)	NA	No	58
Stanford University	2	Female	Pontine diffuse midline glioma	NA	No	1140
Stanford University	3	Male	Pontine diffuse midline glioma	XRT	No	487
Stanford University	4	Male	Pontine diffuse midline glioma	XRT	No	196
Stanford University	6	Female	Pontine diffuse midline glioma	XRT	No	223
Stanford University	11	Female	Pontine diffuse midline glioma	XRT, Vorinostat	No	Censored at 201
Stanford University	7	Female	Pontine diffuse midline glioma	XRT	No	Censored at 5
Stanford University	10	Male	Pontine diffuse midline glioma, H3WT	XRT (only 4 doses)	No	66
Stanford University	3	Male	Pontine diffuse midline glioma, H3.1K27M, H3F3A gain, MDM4 gain, ACVR1 point mutation, NTRK1 gain, CDK18 gain, CLK1 gain, CLK2 gain, CLK4 point mutation, NUAK2 gain, STK36 gain, PPP1CB gain, MAPKAPK2 gain	XRT, Rindopepimut, GM-CSF	No	471
Stanford University	11	Male	Pontine diffuse midline glioma	XRT, TMZ, Veliparib	No	484
Stanford University	13	Female	Medulla diffuse midline glioma	XRT, TMZ, Rindopepimut, GM-CSF, imetelstat	No	245
Stanford University	6	Female	Pontine diffuse midline glioma	XRT, TMZ, Rindopepimut, GM-CSF	No	290
Stanford University	8	Male	Cervical glioblastoma (glioblastoma multiforme, astrocytoma WHO IV)	XRT, TMZ, Subtotal Resection	No	894
Stanford University	3	Male	Pontine diffuse midline glioma	XRT	No	Censored at 105
Stanford University	12	Female	Pontine diffuse midline glioma	XRT	No	195

Stanford University	8	Male	Pontine diffuse midline glioma	XRT, Bevacizumab, Imetelstat	No	384
Stanford University	8	Female	Pontine diffuse midline glioma	XRT, cabazitaxel	No	277
Stanford University	9	Female	Pontine diffuse midline glioma, H3K27-altered	NA	No	78
Stanford University	2	Male	Pontine diffuse midline glioma	XRT, cabazitaxel	No	558
Stanford University	4	Female	Pontine diffuse midline glioma, H3K27-altered	XRT, TMZ, Bevacizumab	No	Censored at 645
Stanford University	10	Female	Pontine diffuse midline glioma	XRT, TMZ, ABT-888	Yes (1 dose)	235
Stanford University	5	Male	Pontine diffuse midline glioma	XRT, TMZ, ABT-888	No	183
Stanford University	6	Male	Pontine diffuse midline glioma	XRT,nivolumab x4 doses	No	Censored at 21
Stanford University	6	Female	Pontine diffuse midline glioma, H3K27-altered	XRT, Subtotal resection, antineoplaston	No	515
Stanford University	2	Male	Pontine diffuse midline glioma	XRT, Bevacizumab	No	Censored at 25
Stanford University	14	Female	Spinal diffuse midline glioma, H3K27-altered	Subtotal Resection, XRT, TMZ, intrathecal liposomal cytarabine, panobinostat	No	198
Stanford University	13	Female	Pontine diffuse midline glioma	XRT, panobinostat	No	350
Stanford University	3	Female	Pontine diffuse midline glioma	XRT	No	Censored at 98
Stanford University	16	Male	Pontine diffuse midline glioma	XRT, panobinostat	No	393
Stanford University	6	Female	Pontine diffuse midline glioma	XRT, panobinostat x1 dose	No	266
Stanford University	6	Male	Pontine diffuse midline glioma, H3K27-altered	XRT, TMZ, Subtotal resection, CCNU, pomalidomide, oral cyclophosphamide, oral topotecan	No	395
Stanford University	8	Male	Pontine diffuse midline glioma, H3K27-altered	XRT, panobinostat	No	176
Stanford University	2	Male	Pontine diffuse midline glioma	XRT	No	385

Stanford University	6	Male	Pontine diffuse midline glioma	XRT	No	Censored at 39
Stanford University	7	Male	Pontine diffuse midline glioma, H3K27-altered	XRT	No	185
Stanford University	5	Male	Pontine diffuse midline glioma, H3K27-altered	XRT	No	Censored at 187
Stanford University	10	Male	Pontine diffuse midline glioma	XRT, ONC201	No	190
Stanford University	2	Male	Pontine diffuse midline glioma	XRT	No	462
Stanford University	5	Male	Pontine diffuse midline glioma, H3WT, TP53 loss and point mutation, PDGFR loss, HDAC3 loss, CDK1 gain, CLK4 loss, MAPK7 loss, MYLK loss, TGFBR2 loss, GSK3B loss, PSMB5 gain, PIK3CB loss, HSPBAP1 loss, MDM2 gain, AURKB loss	XRT (only two fractions)	No	217
Stanford University	1	Male	Pontine diffuse midline glioma	XRT, Cisplatin, Cyclophosphamide, Etoposide, Vincristine	No	792
Stanford University	4	Female	Pontine diffuse midline glioma	XRT, Etoposide	No	410
Stanford University	8	Female	Pontine diffuse midline glioma	XRT, Etoposide, Topotecan	No	646
Stanford University	9	Female	Pontine diffuse midline glioma	XRT, TMZ, Etoposide, Thalidomide	No	514
Stanford University	5	Male	Pontine diffuse midline glioma	XRT	No	230
Stanford University	5	Female	Pontine diffuse midline glioma	XRT	No	255
Stanford University	4	Female	Pontine diffuse midline glioma	XRT, Cyclosporine, Etoposide, Vincristine	No	291
Stanford University	5	Female	Pontine diffuse midline glioma	XRT	No	255
Stanford University	4	Male	Pontine diffuse midline glioma	XRT	No	314
Stanford University	6	Female	Pontine diffuse midline glioma	XRT, Cyclosporine, Etoposide, Vincristine	No	222
Stanford University	5	Female	Pontine diffuse midline glioma	XRT, Etoposide, Vincristine	No	295
Stanford University	5	Male	Pontine diffuse midline glioma	XRT, Etoposide	No	305

Stanford University	11	Male	Pontine diffuse midline glioma	XRT, Etoposide, Vincristine	No	115
Stanford University	6	Female	Pontine diffuse midline glioma	XRT, Etoposide, Vincristine	No	218
Stanford University	5	Male	Pontine diffuse midline glioma	XRT, Gadolinium texaphyrin	No	273
Stanford University	4	Female	Pontine diffuse midline glioma	XRT, TMZ	No	190
Stanford University	5	Female	Pontine diffuse midline glioma	XRT, TMZ	No	302
Stanford University	6	Female	Pontine diffuse midline glioma	XRT	No	336
Stanford University	8	Female	Pontine diffuse midline glioma	XRT	No	434
Stanford University	7	Female	Pontine diffuse midline glioma	XRT, Gadolinium texaphyrin	No	353
Stanford University	5	Male	Pontine diffuse midline glioma	XRT	No	160
Stanford University	12	Male	Pontine diffuse midline glioma	XRT	No	Censored at 225
Stanford University	3	Male	Pontine diffuse midline glioma, H3K27-altered	XRT, panobinostat	No	Censored at 427

Legend:
XRT = radiation
TMZ = temozolomide
COG = Children’s Oncology Group

Supplementary Table 2.

Institution	Age (years)	Sex	Diagnosis	Other treatments	Levetiracetam yes/no	Overall survival (days)
University of Michigan	0.6	Female	Glioblastoma (glioblastoma multiforme, astrocytoma WHO IV), SETD2 S396* mutation, PDGFB amplification (19 copies), H3WT	XRT, TMZ, cisplatin, cyclophosphamide, etoposide, vincristine, dasatinib, Tandem transplant, Subtotal Resection	Yes	871
University of Michigan	16	Male	Cortical/hemispheric astrocytoma, PDGFRA mutant, H3WT	XRT, TMZ, Tripleptal, Vimpat, Dasatanib/Everolimus	Yes	1016
University of Michigan	9	Female	Cortical glioblastoma, TP53 P152L, TP53: Splice donor of exon 4 ATRX R1022* (monoallelic), PRMT2 D95G, Aneuploid CDK4 amplification (chr12; 8 copies), SMARCD1 amplification (chr12; 8 copies), NCOR2 amplification (chr12; 10 copies)	Subtotal resection	Yes	753
University of Michigan	8	Male	Glioblastoma (glioblastoma multiforme, astrocytoma WHO IV), Hypermutation (401.9 Mutations/Mb): TP53 R248W, R158, HDICER1 E1705K, MAP4K3 M1, AMER1 R358*, BCOR R1513*, CTNNA2 R882*, CTNNA1 R451*, IDH1 R100*, KMT2A R407*, PTPN2 R354*, NF1 E2207*, ATM I1581fs, BAX E41fs, FLG E160fs, SOS1 L518fs, NR4A2 Q109fs, KMT2C F835fs, Copy gain chr5 (p15.3-p13.2); Copy loss chr4 (q13.1-q22.1)	XRT, proton XRT, TMZ, bevacuzimab, vorinostat, Valproic acid, cytox, doxorubicin, vincristine, ara-c, methotrexate, pembrolizumab, Subtotal Resection	Yes	3285
University of Michigan	18	Female	Anaplastic pleomorphic xanthoastrocytoma, BRAF V600ETP53 AC276GG, NUTM2F R176G, IRS4 Y828*, PKHD1 A3742V, Genome-wide polyploidy CDKN2A/2B homozygous deletion	Proton XRT, Debrafenib, trametinib, Subtotal Resection	Yes	Censored at 959
University of Michigan	15	Male	Anaplastic astrocytoma, IDH1 R123H-negative, 17.7 Mutations/Mb Microsatellite instability (MSI) signature noted TP53 R273C, PTPN11 A72V, PIK3CA Q546R,	Proton XRT, Pembrolizumab, Bevacuzimab, vimpat	Yes	480

			MITF R110* stopgain, NF1 D2346fs, NF1 G675fs, MSH3 K383fs, WRN E510del, Additional frame-shift mutations: CBX5 K106fs, PDS5B K1318fs, SETD1B H8fs, DDR1 P483fs, TET1 K23fs, SMC5 I939fs, SETD2 N1396fs, TET3 N351fs, copy loss chr14; copy gain chr17q			
University of Michigan	15	Male	Diffuse astrocytoma	XRT, TMZ, Everolimus, Lacosamide	Yes	233
University of Michigan	11	Male	Anaplastic astrocytoma, TP53 I195T, NF1 R440* stopgain, ERBB2 K831T, SCN5A E1060* stopgain, E1070Q, DOCK2 T1279M, ECT2L R233P, Amplifications: Chr3 (CTNNB1, ETV5, SOX2); chr13 (IRS2)	XRT, TMZ, Depakote	Yes	82
University of Michigan	18	Male	Anaplastic astrocytoma (astrocytoma WHO III), ATRX R2178G, PTPN11 Q510L, MYH11 R449Q, E2F1 R166C, POU5F1B R230Q, MYB amplification (exon 1 to 9), Copy loss chr1p-ter, 3p, 2p; UPD chr11p	Vimpat, Gross Total Resection	Yes	Censored at 572
University of Michigan	16	Female	Glioblastoma (glioblastoma multiforme, astrocytoma WHO IV), H3.3 G34R, ATRX E1461* stopgain, GRM3 L91F, PDGFRA P250S, TP53 D186fs insertion, Aneuploidy (polyploidy)	XRT, TMZ, lomustine, Subtotal Resection	Yes	Censored at 309
University of Michigan	18	Male	Anaplastic astrocytoma (astrocytoma WHO III), ATRX S1379* stopgain, NRG3 P289H, CDKN2A/2B deletion, PTPRM deletion, PTPN2 deletion, Copy loss: chr1p, 5q, 11q, 13, 14 UPD: chr17p (TP53), chr4q, 20p	XRT, TMZ, Irinotecan, Palbociclib, Subtotal Resection	Yes	165
University of Michigan	1	Female	Anaplastic astrocytoma (astrocytoma WHO III)	Larotrectinib, Vigabatrin	Yes	Censored at 169
Stanford University	6	Female	Thalamic and medial temporal lobes anaplastic astrocytoma (astrocytoma WHO III)	XRT, TMZ, Lomustine	Yes	277
Stanford University	8	Female	Anaplastic astrocytoma (astrocytoma WHO III)	XRT, TMZ	Yes	404

Stanford University	12	Female	Anaplastic astrocytoma (astrocytoma WHO III)	XRT, TMZ, Topotecan, oral cyclophosphamide (5 days)	Yes	571
Stanford University	11	Female	Glioblastoma (glioblastoma multiforme, astrocytoma WHO IV)	Gross Total Resection	Yes	31
Stanford University	20	Male	Glioblastoma (glioblastoma multiforme, astrocytoma WHO IV)	XRT, TMZ, Bevacizumab	Yes	360
Stanford University	8	Male	Anaplastic astrocytoma (astrocytoma WHO III)	XRT, TMZ, Bevacizumab	Yes	262
Stanford University	15	Male	Glioblastoma (glioblastoma multiforme, astrocytoma WHO IV)	XRT, TMZ, Celocoxib, Lapatinib, Rindopepimut, GM-CSF, Subtotal Resection	Yes	649
Stanford University	7	Male	Glioblastoma (glioblastoma multiforme, astrocytoma WHO IV)	XRT, TMZ, Bevacizumab, Cyclophosphamide, Gross Total Resection	Yes	750
Stanford University	0.1	Female	Glioblastoma (glioblastoma multiforme, astrocytoma WHO IV)	Subtotal resection, TMZ, Carboplatin, Cisplatin, Cyclophosphamide, Eroltinib, Etoposide, Methotrexate, Topotecan, Vincristine, Bevacizumab, Irinotecan	Yes	Censored at 3529
Stanford University	17	Male	Glioblastoma (glioblastoma multiforme, astrocytoma WHO IV)	XRT, TMZ, Bevacizumab	Yes	432
Stanford University	15	Male	Glioblastoma (glioblastoma multiforme, astrocytoma WHO IV)		Yes	Censored at 38
Stanford University	2	Male	Glioblastoma (glioblastoma multiforme, astrocytoma WHO IV), H3WT	Carboplatin, Cisplatin, Cyclophosphamide, Erlotinib, Etoposide, Methotrexate, Topotecan, Vincristine, Gross Total Resection	Yes	Censored at 2504
Stanford University	24	Female	Glioblastoma (glioblastoma multiforme, astrocytoma WHO IV)	XRT, TMZ, Bevacizumab, Cyberknife, Carmustine, Erlotinib, Gross Total Resection	Yes	525
Stanford University	2	Male	Glioblastoma (glioblastoma multiforme, astrocytoma WHO IV)	Subtotal Resection	Yes	107
Stanford University	12	Female	Glioblastoma (glioblastoma multiforme, astrocytoma WHO IV)	XRT, TMZ, Bevacizumab, etoposide, Subtotal Resection	Yes	709
Stanford University	1	Male	Glioblastoma (glioblastoma multiforme, astrocytoma WHO IV)	Carboplatin, Cisplatin, Cyclophosphamide, Eroltinib, Etoposide, Methotrexate, Topotecan, Vincristine, Gross Total Resection	Yes	Censored at 2284

Stanford University	10	Female	Anaplastic astrocytoma (astrocytoma WHO III)	XRT, TMZ, Bevacizumab, Carmustine, Irinotecan	Yes	Censored at 1093
Stanford University	18	Female	Glioblastoma (glioblastoma multiforme, astrocytoma WHO IV)	XRT, TMZ	Yes	122
Stanford University	12	Female	Glioblastoma (glioblastoma multiforme, astrocytoma WHO IV)	XRT, Cisplatin, Cyclophosphamide, Etoposide, Vincristine, cabazitaxel, Subtotal resection	Yes	Censored at 1112
Stanford University	0.3	Male	Glioblastoma (glioblastoma multiforme, astrocytoma WHO IV)	Cisplatin, Cyclophosphamide, Vincristine, topotecan, erlotinib, Gross Total Resection	Yes	Censored at 1210
Stanford University	15	Male	Glioblastoma (glioblastoma multiforme, astrocytoma WHO IV)	XRT, TMZ, CCNU, Bevacizumab, subtotal resection	Yes	732
Stanford University	7	Male	Anaplastic astrocytoma (astrocytoma WHO III)	XRT, TMZ, Bevacizumab, Optune, Irinotecan, Subtotal Resection	Yes	1360
Stanford University	18	Female	Glioblastoma (glioblastoma multiforme, astrocytoma WHO IV)	XRT, TMZ, Bevacizumab, Erlotinib, Near Total Resection	Yes	312
Stanford University	3	Male	Pilocytic astrocytoma (WHO I), relapsed as anaplastic astrocytoma (WHO III)	XRT, TMZ, Bleomycin, P-32, gross total resection	Yes	Censored at 9280
Stanford University	17	Female	Anaplastic astrocytoma (astrocytoma WHO III)	XRT, subtotal resection, TMZ, Bevacizumab, Irinotecan	Yes	1033
Stanford University	11	Female	Anaplastic astrocytoma (astrocytoma WHO III)	XRT, TMZ, Etoposide, Irinotecan, Oxaliplatin, Subtotal Resection	Yes	866
Stanford University	0.4	Female	Glioblastoma (glioblastoma multiforme, astrocytoma WHO IV), H3WT	Antineoplastons, etoposide, Bevacizumab, Gross Total Resection	Yes	Censored at 432
Stanford University	20	Female	Glioblastoma (glioblastoma multiforme, astrocytoma WHO IV)	XRT, TMZ, Gross Total Resection	Yes	Censored at 70
University of Michigan	14	Male	Right Parietal Glioblastoma Multiforme, somatic TSC2 R611W mutation, homozygous deletion of PAK1, germline loss of TP53	Proton XRT, everolimus, Gross total resection	No	Censored at 1953
University of Michigan	16	Female	Glioblastoma multiforme, PDGFRRA V536E and P577R, STAG2 R263W	XRT, dasatinib, etoposide, subtotal resection	No	Censored at 203
University of Michigan	15	Male	Pleomorphic, xanthoastrocytoma, grade 2, BRAF V600E positive	Proton XRT, subtotal resection	No	Censored at 836
University of Michigan	7	Female	Anaplastic ependymoma, PF-EPN-A, 1q and 5p gain	Proton XRT, Gross total resection	No	Censored at 811
Stanford University	20	Male	Anaplastic astrocytoma (astrocytoma WHO III)	XRT, Carboplatin, Lomustine, Procarbazine, Vincristine	No	417

Stanford University	10	Male	Anaplastic astrocytoma (astrocytoma WHO III)	XRT, TMZ, Etoposide, Thalidomide, Subtotal Resection	No	252
Stanford University	0.8	Male	Glioblastoma (glioblastoma multiforme, astrocytoma WHO IV)	Etoposide, Subtotal Resection	No	146
Stanford University	16	Male	Anaplastic astrocytoma (astrocytoma WHO III)	XRT, TMZ, Subtotal Resection	No	Censored at 57
Stanford University	17	Male	Glioblastoma (glioblastoma multiforme, astrocytoma WHO IV)	XRT, TMZ, Oxaliplatin, Subtotal Resection	No	Censored at 386
Stanford University	15	Female	Glioblastoma (glioblastoma multiforme, astrocytoma WHO IV)	XRT, TMZ, Subtotal Resection	No	Censored at 61
Stanford University	2	Female	Anaplastic astrocytoma (astrocytoma WHO III)	Gross Total Resection	No	Censored at 26
Stanford University	17	Female	Anaplastic astrocytoma (astrocytoma WHO III), H3WT	XRT, Subtotal Resection	No	Censored at 411
Stanford University	4	Male	Anaplastic astrocytoma (astrocytoma WHO III)	Gross Total Resection	No	Censored at 32
Stanford University	9	Female	Glioblastoma (glioblastoma multiforme, astrocytoma WHO IV)	XRT, TMZ	No	25
Stanford University	14	Male	Glioblastoma (glioblastoma multiforme, astrocytoma WHO IV)	XRT, TMZ, Lomustine, Subtotal Resection	No	285
Stanford University	0	Male	Glioblastoma (glioblastoma multiforme, astrocytoma WHO IV)	TMZ, Lomustine, Subtotal Resection	No	Censored at 17
Stanford University	17	Female	Thalamic glioblastoma (glioblastoma multiforme, astrocytoma WHO IV)	XRT, TMZ, Lomustine, Bevacizumab	No	837
Stanford University	16	Female	Glioblastoma (glioblastoma multiforme, astrocytoma WHO IV)	XRT, TMZ, Carboplatin, Cisplatin, Cyclophosphamide, Vincristine, Bevacizumab, Irinotecan, Subtotal Resection	No	Censored at 2067
Stanford University	13	Female	Posterior fossa glioblastoma (glioblastoma multiforme, astrocytoma WHO IV)	XRT, TMZ, Bevacizumab, Imatinib mesylate	No	523
Stanford University	11	Female	Glioblastoma (glioblastoma multiforme, astrocytoma WHO IV)	XRT, TMZ, Bevacizumab, Subtotal Resection, BCNU, irinotecan	No	705
Stanford University	10	Male	Glioblastoma (glioblastoma multiforme, astrocytoma WHO IV)	XRT, TMZ, Bevacizumab, Cediranib, Carmustine, Irinotecan, Gross Total Resection	No	518
Stanford University	9	Female	Pontine and cerebellar anaplastic astrocytoma (astrocytoma WHO III)	XRT, Arsenic Trioxide	No	Censored at 220
Stanford University	11	Female	Glioblastoma (glioblastoma multiforme, astrocytoma WHO IV)	XRT, TMZ, Arsenic Trioxide, Bevacizumab, Irinotecan,	No	424

				Cyclophosphamide, subtotal resection		
Stanford University	16	Male	Anaplastic astrocytoma (astrocytoma WHO III), H3WT	XRT, TMZ, Subtotal Resection	No	Censored at 3893
Stanford University	6	Male	Glioblastoma (glioblastoma multiforme, astrocytoma WHO IV)	NA	No	Censored at 12
Stanford University	0	Female	Glioblastoma (glioblastoma multiforme, astrocytoma WHO IV)	Subtotal Resection	No	1
Stanford University	11	Female	Anaplastic astrocytoma (astrocytoma WHO III)	XRT, TMZ, Bevacizumab, Etoposide	No	258
Stanford University	9	Male	Glioblastoma (glioblastoma multiforme, astrocytoma WHO IV)	XRT, TMZ, Bevacizumab	No	333
Stanford University	9	Female	Glioblastoma (glioblastoma multiforme, astrocytoma WHO IV)	XRT, TMZ, Bevacizumab, Lomustine, Gross Total Resection	No	Censored at 331
Stanford University	7	Female	Thalamic anaplastic astrocytoma (astrocytoma WHO III)	XRT, TMZ, Carboplatin, Vincristine	No	Censored at 308
Stanford University	2	Female	Glioblastoma (glioblastoma multiforme, astrocytoma WHO IV), H3WT	XRT, TMZ, Vorinostat, Gross Total Resection	No	Censored at 556
Stanford University	11	Female	Diffuse astrocytoma (astrocytoma WHO II)	Gross Total Resection	No	Censored at 1475
Stanford University	4	Female	Anaplastic astrocytoma (astrocytoma WHO III)	Gross Total Resection	No	Censored at 4830
Stanford University	9	Female	Anaplastic astrocytoma (astrocytoma WHO III)	XRT, TMZ, Lomustine	No	Censored at 249
Stanford University	*in utero* (at 36-week ultrasound)	Female	Glioblastoma (glioblastoma multiforme, astrocytoma WHO IV), H3WT, IDH WT	Etoposide, Carboplatin, Cyclophosphamide, Vincristine	No	Censored at 883
Stanford University	5	Female	Glioblastoma (glioblastoma multiforme, astrocytoma WHO IV), H3K27-altered		No	Censored at 161
Stanford University	2	Male	Mixed malignant germ cell tumor	XRT, Carboplatin, Etoposide, Ifosfamide, Subtotal Resection	No	187
Stanford University	0.6	Male	Anaplastic astrocytoma (astrocytoma WHO III)	Subtotal Resection	No	Censored at 30
Stanford University	0.7	Female	Glioblastoma (glioblastoma multiforme, astrocytoma WHO IV), H3WT	Cisplatin, Cyclophosphamide, Methotrexate, Vincristine	No	Censored at 262

Stanford University	11	Female	Anaplastic ependymoma	XRT, Carboplatin, Cyclophosphamide, Etoposide, Vincristine	No	Censored at 107
Stanford University	2	Female	Anaplastic astrocytoma (astrocytoma WHO III), H3WT	Carboplatin, Vinblastine, Vincristine	No	Censored at 281
Stanford University	7	Female	Anaplastic astrocytoma (astrocytoma WHO III)	XRT, Cisplatin	No	176
Stanford University	6	Male	Anaplastic astrocytoma (astrocytoma WHO III)	XRT, Cisplatin, Subtotal Resection	No	157
Stanford University	9	Male	Glioblastoma (glioblastoma multiforme, astrocytoma WHO IV)	Carmustine, Etoposide, 6-thioguanine, Subtotal Resection	No	86
Stanford University	5	Female	Anaplastic astrocytoma (astrocytoma WHO III)	XRT, Carboplatin, Etoposide, Ifosfamide	No	Censored at 308
Stanford University	10	Male	Glioblastoma (glioblastoma multiforme, astrocytoma WHO IV)	XRT, TMZ, Celocoxib, Subtotal Resection	No	Censored at 53
Stanford University	13	Male	Glioblastoma (glioblastoma multiforme, astrocytoma WHO IV)	XRT, Lomustine, Procarbazine, Vincristine, Etoposide, Subtotal Resection	No	894
Stanford University	17	Female	Glioblastoma (glioblastoma multiforme, astrocytoma WHO IV)	XRT, TMZ, Carmustine, Imatinib mesylate	No	676
Stanford University	17	Female	Glioblastoma (glioblastoma multiforme, astrocytoma WHO IV)	XRT, TMZ	No	293
Stanford University	14	Male	Glioblastoma (glioblastoma multiforme, astrocytoma WHO IV)	XRT, TMZ, Lomustine, Procarbazine, Vincristine, Thalidomide, Subtotal Resection	No	1993
Stanford University	12	Female	Glioblastoma (glioblastoma multiforme, astrocytoma WHO IV)	XRT, Lomustine, Procarbazine, Vincristine, Gross Total Resection	No	Censored at 4976
Stanford University	4	Male	Glioblastoma (glioblastoma multiforme, astrocytoma WHO IV)	Subtotal Resection	No	51
Stanford University	13	Male	Astrocytoma (anaplastic vs pleomorphic xanthoastrocytoma)	XRT, TMZ, Gross Total Resection	No	Censored at 3007
Stanford University	7	Female	Anaplastic astrocytoma (astrocytoma WHO III)	XRT, Carmustine, Cisplatin, Topotecan, Subtotal Resection	No	347
Stanford University	1	Female	Anaplastic astrocytoma (astrocytoma WHO III)	XRT, TMZ, Thalidomide	No	3852
Stanford University	2	Female	Gliosarcoma	XRT, Carmustine, O-6-benzylguanine, Subtotal Resection	No	222
Stanford University	8	Female	Anaplastic astrocytoma (astrocytoma WHO III)	XRT, TMZ, Carmustine, O-6-benzylguanine	No	279

Stanford University	2	Male	Diffuse astrocytoma (WHO II), then glioblastoma (glioblastoma multiforme, astrocytoma WHO IV)	XRT, TMZ, Subtotal Resection, bevacizumab, regorafenib, palbociclib, savolitinib	No	6957
Stanford University	7	Male	Anaplastic astrocytoma (astrocytoma WHO III)	XRT, TMZ, Imatinib mesylate, Subtotal Resection	No	263

Legend:
XRT = radiation
TMZ = temozolomide
