# Supplementary material for: Levetiracetam therapeutically targets GABAergic synapses in diffuse midline glioma
Source: Nat Med. 2026 Sep 17;32(10):3792–800. doi: 10.1038/s41591-026-04646-6 (PMC13668697; doi:10.1038/s41591-026-04646-6)
Supplement: Supplementary file 2 [file 41591_2026_4646_MOESM2_ESM.pdf]

nature portfolio

Corresponding author(s): Michelle Monje

Last updated by author(s): May 20, 2026

Reporting Summary

Nature Portfolio wishes to improve the reproducibility of the work that we publish. This form provides structure for consistency and transparency in reporting. For further information on Nature Portfolio policies, see our Editorial Policies and the Editorial Policy Checklist.

Statistics

For all statistical analyses, confirm that the following items are present in the figure legend, table legend, main text, or Methods section.

n/a	Confirmed	
☐	☒	The exact sample size (n) for each experimental group/condition, given as a discrete number and unit of measurement
☐	☒	A statement on whether measurements were taken from distinct samples or whether the same sample was measured repeatedly
☐	☒	The statistical test(s) used AND whether they are one- or two-sided<br>*Only common tests should be described solely by name; describe more complex techniques in the Methods section.*
☐	☒	A description of all covariates tested
☐	☒	A description of any assumptions or corrections, such as tests of normality and adjustment for multiple comparisons
☐	☒	A full description of the statistical parameters including central tendency (e.g. means) or other basic estimates (e.g. regression coefficient) AND variation (e.g. standard deviation) or associated estimates of uncertainty (e.g. confidence intervals)
☐	☒	For null hypothesis testing, the test statistic (e.g. F, t, r) with confidence intervals, effect sizes, degrees of freedom and P value noted<br>*Give P values as exact values whenever suitable.*
☒	☐	For Bayesian analysis, information on the choice of priors and Markov chain Monte Carlo settings
☒	☐	For hierarchical and complex designs, identification of the appropriate level for tests and full reporting of outcomes
☒	☐	Estimates of effect sizes (e.g. Cohen's d, Pearson's r), indicating how they were calculated

Our web collection on statistics for biologists contains articles on many of the points above.

Software and code

Policy information about availability of computer code

Data collection	Electrophysiology data were collected with a MultiClamp 700B amplifier (Molecular Devices) and digitzed at 10 kHz with an Axon Digidata 1550B (Molecular Devices) or an InstruTECH LIH 8+8 data acquisition device (HEKA). Data were recorded and analyzed using pClamp 11 software suite (Molecular Devices), AxoGraph X (AxoGraph Scientific) and/or IGOR Pro 8 (Wavemetrics). Confocal images were acquired on either a Zeiss LSM700, Zeiss Airyscan1 LSM800, or Zeiss Airyscan2 LSM980 using Zen 2011 v8.1.
Data analysis	Statistical tests were conducted using GraphPad Prism v9.1.0 software. Confocal images were analyzed and quantified using ImageJ v.2.1.0/1.53c.

For manuscripts utilizing custom algorithms or software that are central to the research but not yet described in published literature, software must be made available to editors and reviewers. We strongly encourage code deposition in a community repository (e.g. GitHub). See the Nature Portfolio guidelines for submitting code & software for further information.

Data

Policy information about availability of data

All manuscripts must include a data availability statement. This statement should provide the following information, where applicable:

- Accession codes, unique identifiers, or web links for publicly available datasets
- A description of any restrictions on data availability
- For clinical datasets or third party data, please ensure that the statement adheres to our policy

All data are available in the manuscript or from the corresponding author upon reasonable request. Source data will be uploaded with the final version of the manuscript.

Research involving human participants, their data, or biological material

Policy information about studies with human participants or human data. See also policy information about sex, gender (identity/presentation), and sexual orientation and race, ethnicity and racism.

Reporting on sex and gender	Sex is reported in Table 1. Both male and female patients were included equally.
Reporting on race, ethnicity, or other socially relevant groupings	N/A
Population characteristics	Age and tumor characteristics are reported in Table 1
Recruitment	retrospective clinical data including all pediatric patients with high-grade gliomas treated at Stanford and University of Michigan
Ethics oversight	IRB approval was also obtained for retrospective analyses of real-world clinical data kept in IRB-approved databases at Stanford University, University Michigan and UCSF. For the cohort from University Medical Center Hamburg-Eppendorf, informed written consent was obtained from all patients or their legal guardians, and further data processing was approved by the medical ethics committee of the Hamburg Chamber of Physicians (PV4904).

Note that full information on the approval of the study protocol must also be provided in the manuscript.

Field-specific reporting

Please select the one below that is the best fit for your research. If you are not sure, read the appropriate sections before making your selection.

☒ Life sciences ☐ Behavioural & social sciences ☐ Ecological, evolutionary & environmental sciences

For a reference copy of the document with all sections, see nature.com/documents/nr-reporting-summary-flat.pdf

Life sciences study design

All studies must disclose on these points even when the disclosure is negative.

Sample size	At least three mice for in vivo experiments, and at least three independent coverslips for in vitro experiments, were used per test group to attain 80% power to detect an effect size of 20% at significance level 0.05.
Data exclusions	No data were excluded from analyses.
Replication	The number and type of biological replicates is indicated in the figure legends and was three or greater for all experiments. At least three independent coverslips or at least three mice were used for each experiment.
Randomization	Mice were randomly assigned to treatment or control groups for all levetiracetam treatment experiments.
Blinding	Imaging and quantification of histological experiments were performed by blinded experimenters. Quantification of electrophysiological data was performed within the same cell before and after treatment and therefore not blinded.

Reporting for specific materials, systems and methods

We require information from authors about some types of materials, experimental systems and methods used in many studies. Here, indicate whether each material, system or method listed is relevant to your study. If you are not sure if a list item applies to your research, read the appropriate section before selecting a response.

Materials & experimental systems

n/a	Involved in the study
☐	☒ Antibodies
☐	☒ Eukaryotic cell lines
☒	☐ Palaeontology and archaeology
☐	☒ Animals and other organisms
☐	☒ Clinical data
☒	☐ Dual use research of concern
☒	☐ Plants

Methods

n/a	Involved in the study
☒	☐ ChIP-seq
☒	☐ Flow cytometry
☒	☐ MRI-based neuroimaging

Antibodies

Antibodies used

Primary antibodies: chicken anti-GFP (1:500, ab1397, Abcam), mouse anti-human nuclei clone 235-1 (1:100, MAB1281, Millipore), rabbit anti-Ki67 (1:500, ab15580, Abcam), chicken anti-neurofilament-H/M (1:500, Antibodies Incorporated, AB_2313554) and rabbit anti-SV2A (1:500, Synaptic Systems, 119 003).

Secondary antibodies: Alexa 488 donkey anti-chicken IgG (703-545-155), Alexa 594 donkey anti-mouse IgG (715-585-150), Alexa 594 donkey anti-rabbit IgG (711-585-152), Alexa 647 donkey anti-mouse IgG (715-605-151), Alexa 647 donkey anti-rabbit IgG (711-605-152), Alexa 647 donkey anti-chicken IgG (703-605-155), at 1:500 (Jackson ImmunoResearch).

Validation

All antibodies have been validated either in the literature and/or by manufacturers for use in mouse immunohistochemistry. To further validate the antibodies in our hands, we confirmed each antibody had staining in the expected cellular patterns and brain-wide distributions.
Antibody validation information: chicken anti-GFP (ab13970, Abcam; Reactivity: Species independent. Manufacturer validation: ICC: GFP-transfected NIH/3T3 Mouse embryo fibroblast cell line); mouse anti-human nuclei clone 235-1 (MAB1281, Millipore; Reactivity: human only, Manufacturer validation: IF neural stem cells transplanted into rat brain); rabbit anti-Ki67 (ab15580, Abcam; Reactivity: mouse, human. Manufacturer validation: IF in Mef1 and HeLa cultures, human skin tissue); chicken anti-neurofilament-H/M (AB_2313554, Antibodies Incorporated; Reactivity: human, mouse, and rat. Manufacturer validation: IF in cultured mouse cortical neurons) and rabbit anti-SV2A (119 003, Synaptic Systems; Reactivity: human, rat, mouse, Guinea pig, cow, hamster, chicken, zebrafish. Manufacturer validation: IF in mouse hippocampal neurons in culture and slice).

Eukaryotic cell lines

Policy information about cell lines and Sex and Gender in Research

Cell line source(s)

The eukaryotic cell cultures used are derived from an electroporation-induced genetic mouse model of H3K27M-DMG (MADR), or are patient-derived cultures of high-grade gliomas generated in the Monje lab from biopsy (SU-pcGBM2) or autopsy tissue (SU-DIPG-VI, SU-DIPGXIII-FL, SU-DIPG36, SU-DIPG50, SU-DIPGXIII-P), or provided by collaborators from patient-derived cultures (SF0232, SF0238). Additionally, human induced pluripotent stem cell (iPSC)–derived GABAergic neurons (Bit.bio io1003) were used for neuron-glioma co-culture assays.

Authentication

Short Tandem Repeat (STR) fingerprinting is performed every 3 months on all cell cultures to ensure authenticity.

Mycoplasma contamination

All cell cultures are routinely tested for mycoplasma contamination and all cultures were tested negative.

Commonly misidentified lines
(See ICLAC register)

No commonly misidentified lines were used.

Animals and other research organisms

Policy information about studies involving animals; ARRIVE guidelines recommended for reporting animal research, and Sex and Gender in Research

Laboratory animals

All in vivo experiments were conducted in accordance with protocols approved by the Stanford University Institutional Animal Care and Use Committee (IACUC) and performed in accordance with institutional guidelines. Animals were housed according to standard guidelines with free access to food and water in a 12 h light:12 h dark cycle. For all xenograft studies, NSG mice (NOD-SCID-IL2R gamma chain-deficient, The Jackson Laboratory) aged 1-6 months were used. For allograft studies, C57BL/6j mice (The Jackson Laboratory) aged 1-6 months were used. Male and female mice were used equally. For brain tumor experiments, the IACUC does not set a limit on maximal tumor volume but rather on indications of morbidity. In no experiments were these limits exceeded as mice were euthanized if they exhibited signs of neurological morbidity or if they lost 15% or more of their body weight.

Wild animals

No wild animals were used.

Reporting on sex

Male and female mice were used equally.

Field-collected samples

No field-collected samples were used.

Ethics oversight	All animal experiments were conducted in accordance with protocols approved by the Stanford University Institutional Animal Care and Use Committee (IACUC) and performed in accordance with institutional guidelines. The IACUC implements regulations from the United States Department of Agriculture (USDA), the Public Health Service (PHS) Policy, California State Regulations and Stanford University Polices and Guidelines to ensure effective and ethical animal research programs.

Note that full information on the approval of the study protocol must also be provided in the manuscript.

Clinical data

Policy information about clinical studies
All manuscripts should comply with the ICMJE guidelines for publication of clinical research and a completed CONSORT checklist must be included with all submissions.

Clinical trial registration	N/A
Study protocol	N/A
Data collection	Retrospective data on patients with high-grade glial tumors were collected from patient databases at Stanford University (1990-2020) and the University of Michigan (2012-2021) and UCSF (2012-2022) through protocols approved by the respective institutional review boards (IRB). Retrospective data were collected from patient records from University Medical Center Hamburg-Eppendorf (Germany) between 2006 and 2021. Informed written consent was obtained from all patients or their legal guardians, and further data processing was approved by the medical ethics committee of the Hamburg Chamber of Physicians (PV4904).
Outcomes	For all clinical data analyses, the outcome measured was overall survival of pediatric high grade glioma patients.

Plants

Seed stocks	*Report on the source of all seed stocks or other plant material used. If applicable, state the seed stock centre and catalogue number. If plant specimens were collected from the field, describe the collection location, date and sampling procedures.*
Novel plant genotypes	*Describe the methods by which all novel plant genotypes were produced. This includes those generated by transgenic approaches, gene editing, chemical/radiation-based mutagenesis and hybridization. For transgenic lines, describe the transformation method, the number of independent lines analyzed and the generation upon which experiments were performed. For gene-edited lines, describe the editor used, the endogenous sequence targeted for editing, the targeting guide RNA sequence (if applicable) and how the editor was applied.*
Authentication	*Describe any authentication procedures for each seed stock used or novel genotype generated. Describe any experiments used to assess the effect of a mutation and, where applicable, how potential secondary effects (e.g. second site T-DNA insertions, mosiacism, off-target gene editing) were examined.*
