# Supplementary material for: Levetiracetam therapeutically targets GABAergic synapses in diffuse midline glioma
Source: Nat Med. 2026 Sep 17;32(10):3792–800. doi: 10.1038/s41591-026-04646-6 (PMC13668697; doi:10.1038/s41591-026-04646-6)
Supplement: Supplementary file 3 [file 41591_2026_4646_MOESM3_ESM.pdf]

nature portfolio

Peer Review File

Levetiracetam therapeutically targets GABAergic synapses in diffuse midline glioma

Corresponding Author: Professor Michelle Monje

This file contains all reviewer reports in order by version, followed by all author rebuttals in order by version.

Version 0:

Reviewer comments:

Reviewer #1

(Remarks to the Author)
Major

Were the pediatric HGG patients analyzed by mutational status to eliminate this as a potential variable accounting for or influencing the levetiracetam treatment effect?

As a control for neurotransmitter specificity, what is the effect on DMG survival if another anti-epileptic drug is analyzed?

How does the effect of levetiracetam on xenograft-bearing mouse survival relative to glutamate receptor antagonist treatment? This is an important control for both comparison and mechanism specificity.

How relevant is a hippocampal slice culture for a diffuse midline brain tumor? While this is a commonly used preparation, is it representative of the brain locations in which these tumors arise and growth?

In the brainstem, is there preferential GABAergic neuron-tumor synapse formation relative to other neuronal subtypes? Rabies tracing might be help elucidate this.

More in-depth analysis of the treated tumors should be included, such as tumor size and contralateral spread.

Minor

The first paragraph in the Introduction seems out of place in introducing the topic of cancer neuroscience, the subject of this report. The authors should consider framing the question differently to focus on pediatric HGG (DMG) and the importance of the tumor microenvironment in treating an otherwise deadly and therapeutically recalcitrant cancer.

Reviewer #2

(Remarks to the Author)
I General comments

Barron et al. investigate the role of levetiracetam on GABAergic synapses in diffuse midline glioma (DMG). They build upon their recent publication (Barron T et al., Nature2025 Mar;639(8056):1060-1068). First, they identify via retrosepctive analyses of real world data of three different institutions that levetiracetam is associated with longer survival in DMG.
Second, they observe reduced cellular growth and proliferation under levetiracetam in preclinical models of DMG.
Third, they find that levetiracetam selectively targets GABAergic neuron-to-glioma synaptic currents in DMGs (and not neuron-to-neuron currents).
The authors conclude:
"Future work, studying larger numbers of patients and stratifying subjects based on DMG location, molecular characteristics, and glioma neuroscience correlative markers will be required to draw conclusions about the potential role of levetiracetam for DMG therapy" (lines 266-269).

The results are novel. The authors give a very thorough description of limitations, particularly of all retrospective analyses. Overall, if these data are further expanded (as suggested by authors in lines 266-269) and prospectively validated in clinical cohorts, this study will have a high significance because it expands the therapeutic options in an area of very high unmet clinical need by a so far un-acknowledged/neglected mechanism.

II Specific comments

1) Retrosepective analyses of three patient cohorts

a) Major comments

- The authors acknowledge the main limitations of these analyses themselves. The retrospective setting is indeed very challenging, particularly if one takes into account that they included patients that had been treated between 1990-2020 (Standford), 2012-2021 (Michigan) and 2006-2021 (Hamburg). It is very demanding to ensure a thorough data collection in a retrospective design and thus this reviewer really acknowledges this effort. How did the authors ensure the collection of a harmonized clinical data set? Did they perform a central pathology review? Did they perform a central imaging review? All these assessments could be performed in a retrospective analyses to increase the comparibility of data. Please add information, data and comment.

- The sample sizes of the three cohorts are very different and it is quite challenging to extract all relevant information from the present manuscript because the information is distributed in the legend and in the text. Please provide a CONSORT-like overview for all three cohorts. Furthermore, the validation cohort is very small, only comprising 19 vs 15 patient as this reviewer understands from Figures 1 e-f. Please expand the number of the validation cohort, e.g. by reaching out to further centers. An increase of sample size for validation plus central review of path/imaging will significantly increase the value of the retrospective analyses and will inform further prospective cohorts.

b) Minor comments

- Lines 292-296: The authors comment on IRB approvals for Stanford and Michigan. Please include information on Hamburg.

2) Preclinical experiments

a) Major comments

- The authors present data from patient-derived Xenograft models. These models are highly informative and helpful to investigate proof-of-concpets and also to investigate human tumors in vivo. As the effect of levetiracetam and basically any other anti-seizure medication is mainly on the brain microenvironment (i.e.: tumor host interaction) and not a pure cell-intrinsic effect, this reviewer recommends further experiments in syngeneic settings for their analyses, e.g. syngeneic murine models and organoid/microtumors ex vivo. Please add experimental data.

- In Section Results and in Figure 2, the authors present survival data from murine xenograft models in panels a, b. Afterwards, they focus on showing effects on proliferation (2c-j) and do not show further survival effects. Furthermore, this reviewer does not see much on the provided "representative confocal images". Please add more survival data, histology data and more assessable images on tumor histology and immunohistochemistry. Did the authors observe any effect on tumor volume?

b) Minor comments

- The number of mice per experimental group varies between the different experiments that are outlined in Figures 2 and 3. Please add the corresponding number in the figure to allow a better and quicker overview at one glance.

3) Tumour subtype-specific mechanism of brain cancer neurophysiology: Effects of levetiracetam on GABAergic neuron-to-glioma versus neuron-to-neuron synatic currents in DMGs

- The experiments presented in Section Results lines 185-228 and in Figure 4 aim at identifying the mechanism of action of levetiracetam on DMG. Using brivaracetam (a more selectively acting anti-seizure medication, i.e. compared with levetiracetam more SV2A-specificity), they conclude that levetiracetam does not act by inhibiting SV2A because they do not observe any effect with brivaracetam. Yet, the dosages that the authors used here for both drugs might effect these different phenotypes, too. Please comment and explain the chosen dosage and its relevance compared with the clinical setting. Furthermore, it would be necessary to perform additional genetic validation by SV2A silencing.

- The main data of this section are shown in Figure 4. The corresponding figure legend indicates the number of cells and mice that had been investigated. These numbers are rather variable. Please comment.

Reviewer #4

(Remarks to the Author)
In this manuscript, the authors report that the approved antiepileptic drug levetiracetam has specific anti-tumor effects in DMG, specific both with respect to tumor entity and antiepileptic drug. This is supported by retrospective patient cohorts from 3 institutions, and two mouse models of DMG. Finally the authors demonstrate data that speaks for a specific influence of LEV on the GABA receptor via a mechanism that still needs to be explored.

This study adds significantly to our understanding of how distinct drugs interfering with neuronal activity can selective influence neuron-to-cancer communications. The authors showed before that GABA-ergic antiepileptic drugs should be avoided, because they fuel tumor growth in DMG; they now demonstrate a tumor cell-specific GABA-inhibitory effect of LEV which is most likely responsible for the antitumor effects (mouse survival and tumor cell proliferaion). The translational / clinical relevance is strong.

The manuscript is very clearly and well written, and the data is presented in a thoughtful and critical way. The methods and statistics appear all adequate. I could find no errors.

Two points should be addressed:

1. Fig. 4: how sure can one be that GABA-ergic synaptic currents have been recorded in the tumor cells? Can the authors modulate the recorded currents in control tumor cells treated with NBQX when a GABA modulator is added?

2. It would be a strong addition to the story if the authors can provide more data on the molecular mechanism of what LEV is exactly modulating in the DMG cells. Can the authors provide gene expression and/or proteomics data? How is GABA signaling exactly influenced (molecularly / electrophysiologically)? Brivaracetam is a much more specific SV2A inhibitor than LEV, implying the possibility that other neurotransmitter receptors or neuronal factors might play in role on the neuronal side.

Version 1:

Reviewer comments:

Reviewer #1

(Remarks to the Author)
No further comments or suggestions

Reviewer #2

(Remarks to the Author)
The authors present a revised version of their study.
They have added new data including a new validation cohort, central neuropath review, a CONSORT chart, extensive further preclinical experiments.
All questions of this reviewer have been adressed.
The clinical data derived from retrospective data sets comprise a rather small set of patients with Diffuse Midline Glioma (DMG) and LEV treatment (n=5 in the validation cohort from Hamburg/Germany, and n=5 from the UCSF validation cohort). How do the authors judge the relevance of clinical data and comparisons of LEV-treated DMG versus non-LEV-treated with these rather small case numbers?

Reviewer #4

(Remarks to the Author)
The authors have responded well to my comments.

Open Access This Peer Review File is licensed under a Creative Commons Attribution 4.0 International License, which permits use, sharing, adaptation, distribution and reproduction in any medium or format, as long as you give appropriate credit to the original author(s) and the source, provide a link to the Creative Commons license, and indicate if changes were made.
In cases where reviewers are anonymous, credit should be given to 'Anonymous Referee' and the source.
The images or other third party material in this Peer Review File are included in the article's Creative Commons license, unless indicated otherwise in a credit line to the material. If material is not included in the article's Creative Commons license and your intended use is not permitted by statutory regulation or exceeds the permitted use, you will need to obtain permission directly from the copyright holder.
To view a copy of this license, visit https://creativecommons.org/licenses/by/4.0/

We were delighted to see the Referees' positive comments and enthusiasm for the manuscript and are grateful for the careful review, helpful suggestions, and insightful questions. We have worked to address the Referees' comments and suggestions in full, which we feel has improved and strengthened the manuscript.

Below, we will present in detail the major changes and then will respond to the Referee comments point-by-point.

Major new findings and additions:

1. We have now added a new validation cohort (form UCSF)
2. We have now performed central neuropathological review for cases with available tissue, and described how the clinical cases were reviewed and verified
3. We have added a new preclinical comparison of levetiracetam to perampanel
4. We have further demonstrated preclinical efficacy, including:
a. Demonstrating improved survival with levetiracetam in a second patient-derived DMG model
b. Testing levetiracetam in a murine (immunocompetent) model of DMG
c. Demonstrating decreased tumor burden (in addition to decreased rate of proliferation)
5. We have added CONSORT diagrams for the main and validation cohorts
6. We have now further demonstrated that SV2A is not the relevant therapeutic target using SV2A knockdown in GABAergic neurons
7. We have added further electrophysiology confirming levetiracetam specifically decreases neuron-to-glioma GABAergic currents, not neuron-to-neuron GABAergic currents

Point-by-point responses

Reviewer #1

We thank the Reviewer for the helpful suggestions.

Major

Were the pediatric HGG patients analyzed by mutational status to eliminate this as a potential variable accounting for or influencing the levetiracetam treatment effect?

In these retrospective datasets dating back as far as 30 years, DMG diagnosis was historically made by MRI imaging alone. This was the standard practice at Stanford until relatively recently. As biopsy became more common, the more recent cases do have molecular analyses which have been added to mutational status of the tumors whenever possible.

As a control for neurotransmitter specificity, what is the effect on DMG survival if another anti-epileptic drug is analyzed?

We have shown in past preclinical work that perampanel, which targets AMPA-type glutamate receptors – another synaptic mechanism promoting DMG growth (Venkatesh et al 2019, *Nature*) shows preclinical benefit in DMG (Venkatesh et al., 2019, *Nature*), and prospective clinical trials are in the planning phases. However, perampanel is not commonly given to children with brain tumors so retrospective analyses are not possible. As shown in the comment below, we have now compared parampanel to levetiracetam and find levetiracetam efficacy superior to parampanel in terms of xenografted mouse overall survival. Anti-seizure drugs that do not target specific neuron-DMG interactions do not show preclinical benefit, as demonstrated here in Figure 3 for phenytoin and ethosuximide:

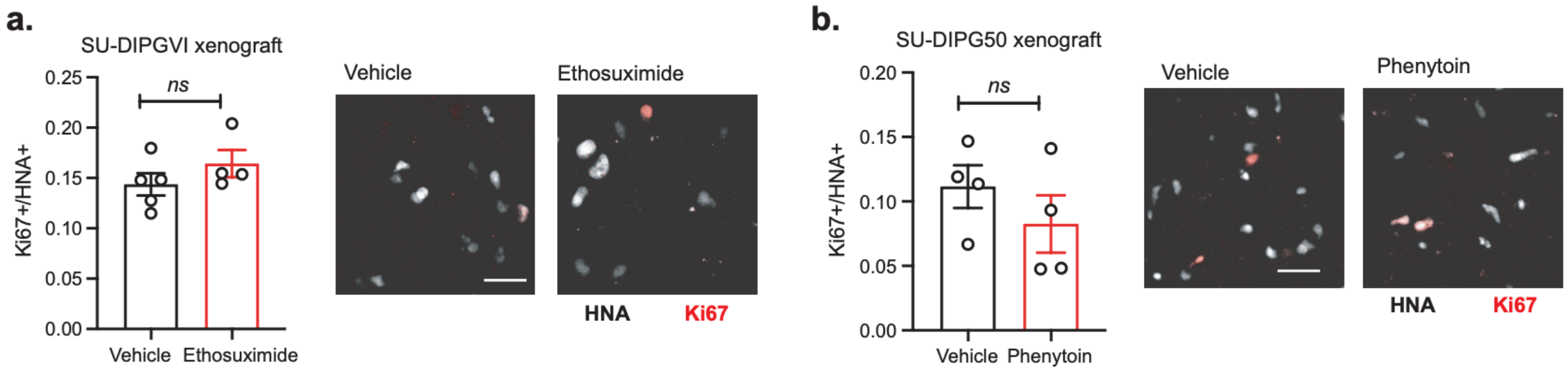

Figure 3. Antiepileptic drugs have variable effects on DMG growth.
a. Ethosuximide treatment in mice with patient-derived SU-DIPGVI xenografts has no effect on cell proliferation (vehicle, n = 5 mice; ethosuximide, n = 4 mice). Representative confocal images of xenografted SU-DIPGVI cells expressing Ki67 (red) and HNA (white; right). Scale bar, 25 µm. Two-tailed Student's t-test.
b. Phenytoin treatment in mice with patient-derived SU-DIPG50 xenografts has no effect on cell proliferation (vehicle, n = 4 mice; phenytoin, n = 4 mice). Representative confocal images of xenografted SU-DIPG50 cells expressing Ki67 (red) and HNA (white; right). Scale bar, 25 µm. Two-tailed Student's t-test.

Analyzing the effect of other anti-epileptic drugs on DMG patient survival in the existing patient cohorts is not feasible, as levetiracetam is by the far the most common agent used in pediatric oncology practice since it is chiefly excreted by the kidney and therefore does not interfere with chemotherapy metabolism.

How does the effect of levetiracetam on xenograft-bearing mouse survival relative to glutamate receptor antagonist treatment? This is an important control for both comparison and mechanism specificity.

We thank the reviewer for bringing up this interesting and important question. We have now performed a survival study showing that levetiracetam increases survival of mice bearing DMG xenografts to a greater extent than the AMPA receptor antagonist perampanel. These new data have been added to Figure 3 and are shown below:

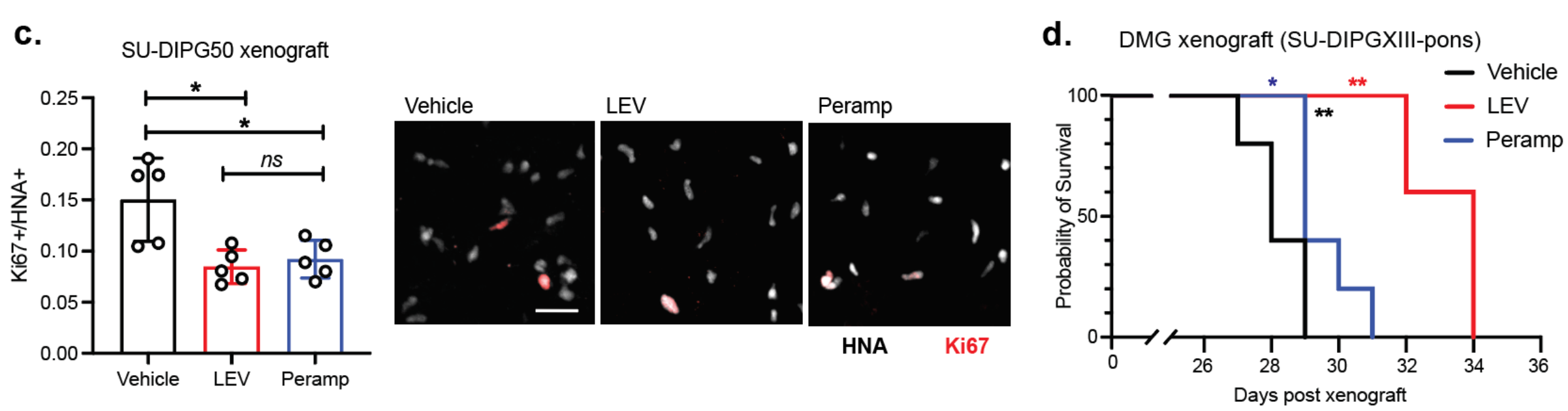

Figure 3. Antiepileptic drugs have variable effects on DMG growth.
c. Effect of levetiracetam (LEV) and perampanel (Peramp) treatment on glioma proliferation in mice with patient-derived DMG xenografts (SU-DIPG50; n = 5 mice for each group), two-tailed Student's t-test. Right, representative confocal images of xenografted SU-DIPG50 cells expressing Ki67 (red) and HNA (white). Scale bar, 25 µm.
d. Kaplan–Meier survival curves of mice with orthotopically xenografted SU-DIPG-XIII-pons tumors treated with LEV, Peramp, or vehicle (n = 5 mice per group).
All data are mean ± s.e.m. *ns*, not significant, $*P < 0.05$, $**P < 0.01$.

How relevant is a hippocampal slice culture for a diffuse midline brain tumor? While this is a commonly used preparation, is it representative of the brain locations in which these tumors arise and growth?

While under-recognized, the hippocampus is often involved in DMG patients. This can be appreciated as medial temporal lobe expansion by MRI (which is typically T2 isointense and non-enhancing), and H3K27M IHC at autopsy frequently shows DMG cells infiltrating the hippocampus in patients. An

example of this is shown in Barron et al, 2025 and replicated below (H3K27M+ nuclei shown by immunohistochemistry in brown; autopsy sample of hippocampus from a 13-year-old female with pontine DMG/DIPG.)

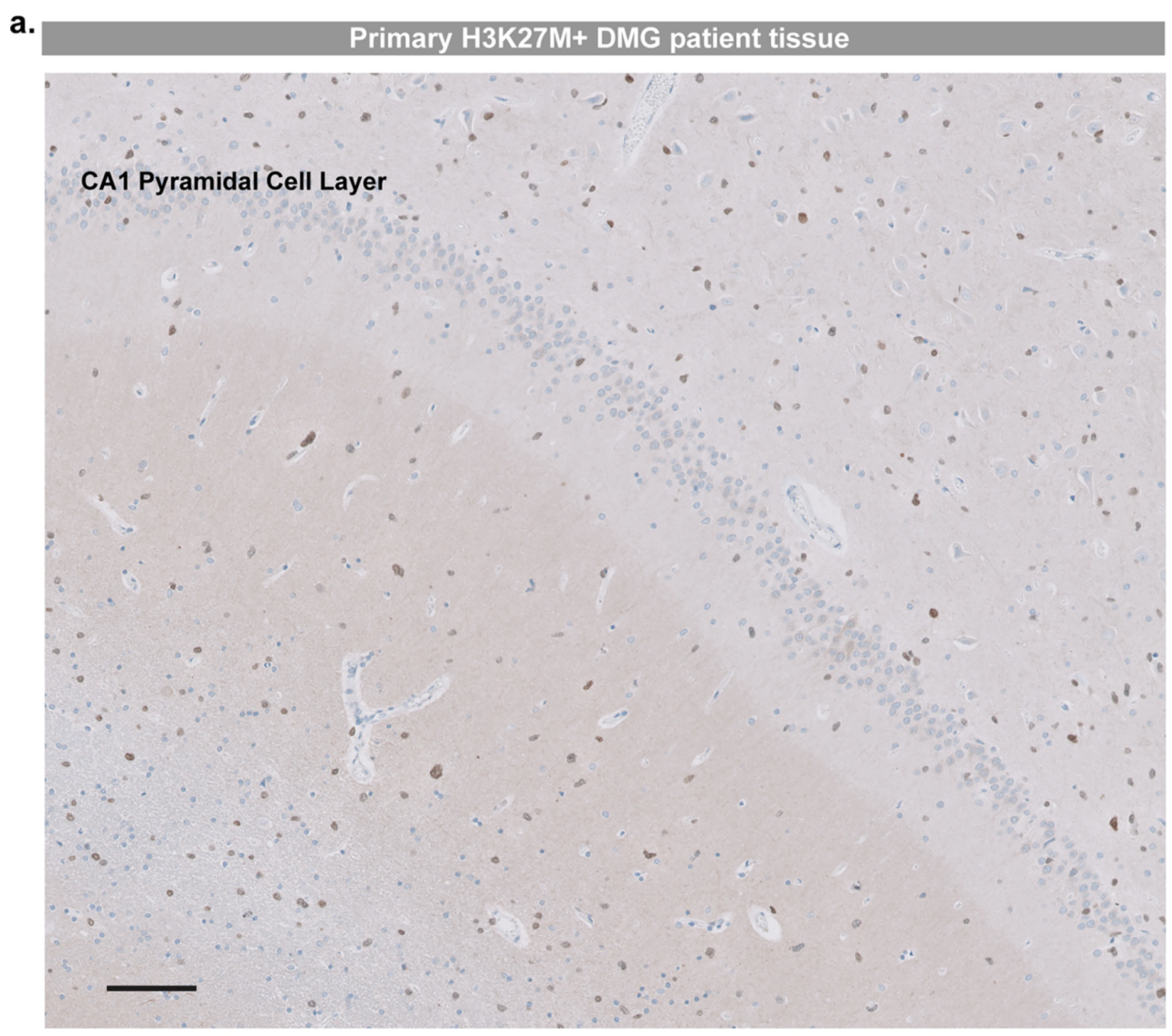

Acute xenografted hippocampal slices were used for electrophysiology experiments due to technical limitations of *in vivo* patch clamp electrophysiology in the brainstem of adult mice. The hippocampus is classically used in patch clamp electrophysiology because it is a well-defined and discrete circuit that results in the most reliable electrophysiology assessments, which is why this approach is used here.

In the brainstem, is there preferential GABAergic neuron-tumor synapse formation relative to other neuronal subtypes? Rabies tracing might be help elucidate this.

The brainstem has many GABAergic neurons, so gliomas in this location may have ample opportunity for GABAergic input. In our previous work (Barron et al., 2025, *Nature*), we xenografted DMG to the brainstem and illustrated that positive allosteric modulation of $GABA_A$ signaling (using the benzodiazepine lorazepam) results in a dose-dependent increase in DMG proliferation rate, accelerated tumor growth, and reduced mouse survival. Importantly, lorazepam only augments GABAergic signaling but does not directly stimulate the $GABA_A$ receptor. These experiments thus demonstrate that GABAergic signaling is occurring and is functionally important in the pons. These data (from Figure 4e-h of Barron et al 2025, *Nature*) are shown below.

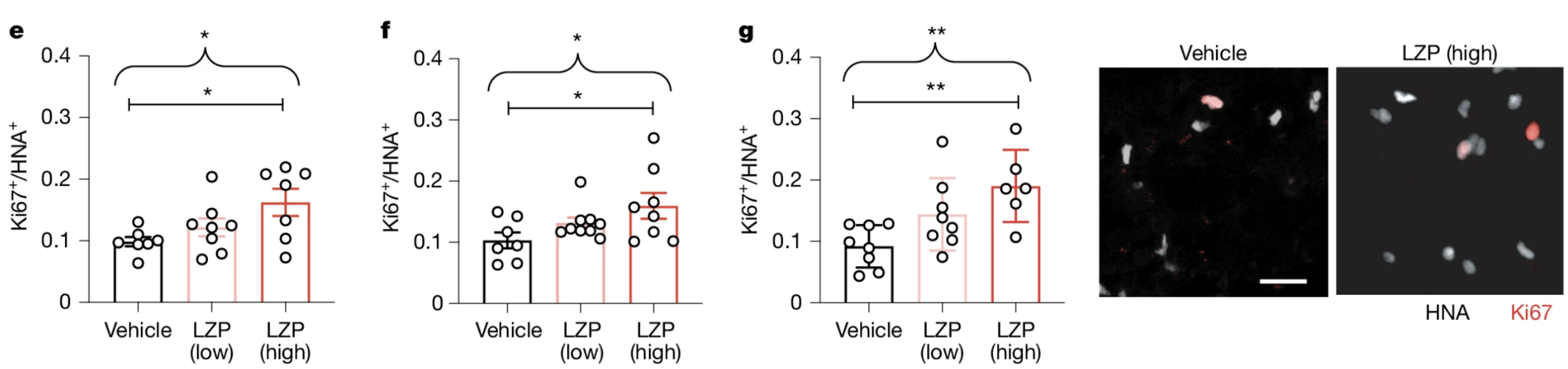

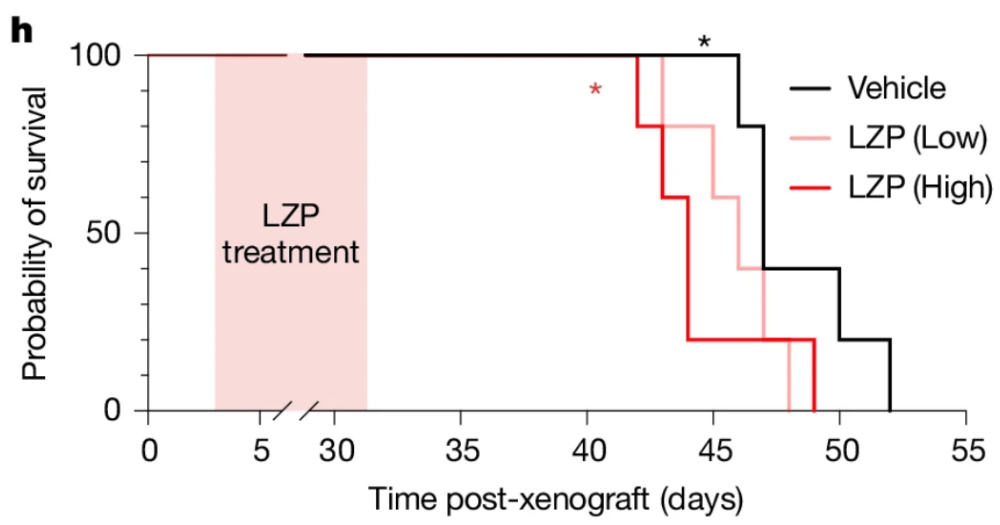

Figure 4. GABAergic signaling drives DMG proliferation.
e-g. Dose-dependent (low=2 mg/kg; high=8 mg/kg) effect of LZP treatment in mice with patient-derived DMG xenografts, SU-DIPGXIII-FL (vehicle, n=7 mice; low, n=8 mice; high, n=7 mice; p=0.0374; **e**), SU-DIPG50 (vehicle, n=7 mice; low, n=9 mice; high, n=8 mice; p=0.0573; **f**), and SU-DIPGVI (vehicle, n=8 mice; low, n=8 mice; high, n=6 mice; p=0.0075; **g**), one-way ANOVA. Straight brackets, Dunnett's multiple comparisons test between two groups (vehicle vs. high; **e**, p=0.0228; **f**, p=0.033; **g**, p=0.0041). Curved brackets, post-test for linear contrast among all groups (**e**, p=0.0124; **f**, p=0.0181; **g**, p=0.002). Right, representative images of xenografted SU-DIPGVI cells in pons expressing Ki67 (red) and HNA (white). Scale bar, 25 µm.
h. Kaplan–Meier survival curves of mice xenografted with patient-derived DMG cells (SU-DIPG-XIII-pons) and treated with LZP or vehicle (n=5 mice per group), demonstrating a significant reduction in survival in mice treated with a high dose of LZP (red star, p=0.0495, Mantel-Cox test), and a dose-dependent reduction in survival (black star, p=0.0320, logrank test for trend).

In response to this important point, we have added discussion of these findings to the manuscript: *"The brainstem, thalamus and spinal cord are rich in GABAergic inputs, and the importance of GABAergic signaling in brainstem DMG is underscored by the tumor growth-promoting effects of lorazepam – which positively modulates but does not directly stimulate GABA receptors - on pontine DMG growth {Barron 2025}."*

Rabies tracing is outside the scope of this paper, but we have recently published the importance of cholinergic synapses in brainstem DMG (Drexler et al., 2025 *Cell*) and serotonergic inputs to brainstem DMG (Drexler et al., 2025 *BioRxiv*). Thus, the circuit inputs to DMG in the brainstem and complex and multiple, but GABAergic inputs are one important component that we can target using re-purposed levetiracetam.

More in-depth analysis of the treated tumors should be included, such as tumor size and contralateral spread.

We thank the reviewer for this excellent suggestion. We have now included additional histological analysis of the tumors and found that there was a decrease in overall tumor burden in levetiracetam-treated mice, but we did not find a significant change in extent of tumor spread. We have added these analyses to Figure 2 and are also shown below:

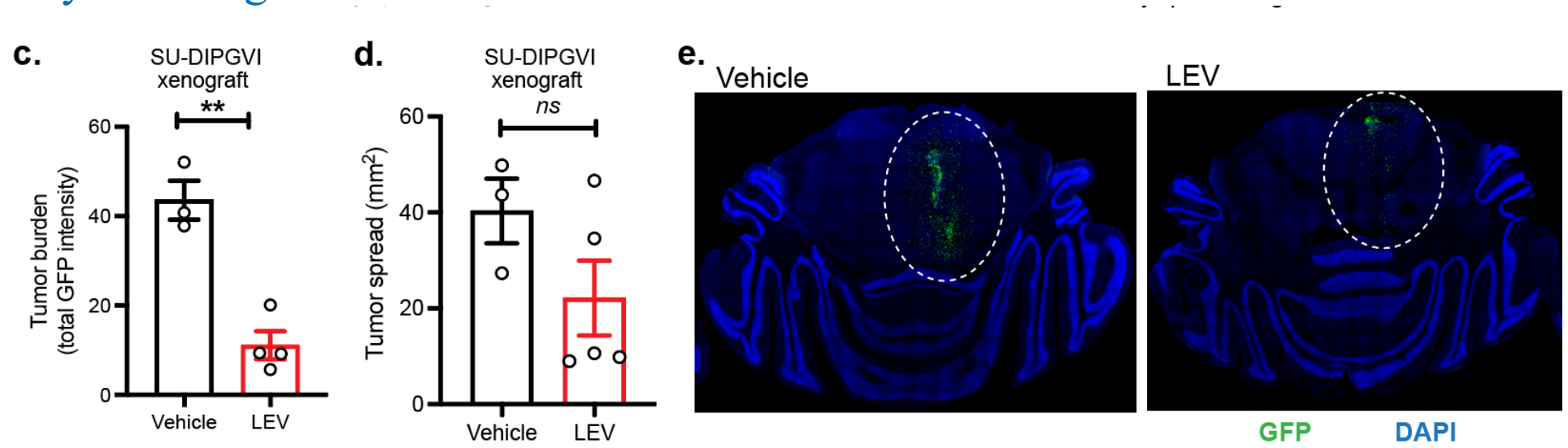

Figure 2. Levetiracetam extends survival and reduces glioma growth in DMG but not hemispheric HGG preclinical models.
c-d. Effect of LEV on overall tumor burden (**c.**) and tumor spread (**d.**) in mice with patient-derived DMG xenografts (SU-DIPGVI; vehicle, n = 3; LEV, n = 5), two-tailed Student's t-test.
e. Representative confocal images of xenografted SU-DIPGVI cells (green) in the pons of mice treated with vehicle or LEV. White dotted line represents area of tumor spread.

Minor

The first paragraph in the Introduction seems out of place in introducing the topic of cancer neuroscience, the subject of this report. The authors should consider framing the question differently to focus on pediatric HGG (DMG) and the importance of the tumor microenvironment in treating an otherwise deadly and therapeutically recalcitrant cancer.
Thank you for this helpful suggestion – we have now edited the introduction accordingly.

Reviewer #2

I General comments

Barron et al. investigate the role of levetiracetam on GABAergic synapses in diffuse midline glioma (DMG). They build upon their recent publication (Barron T et al., Nature 2025 Mar;639(8056):1060-1068). First, they identify via retrospective analyses of real world data of three different institutions that levetiracetam is associated with longer survival in DMG.
Second, they observe reduced cellular growth and proliferation under levetiracetam in preclinical models of DMG.
Third, they find that levetiracetam selectively targets GABAergic neuron-to-glioma synaptic currents in DMGs (and not neuron-to-neuron currents).
The authors conclude:
"Future work, studying larger numbers of patients and stratifying subjects based on DMG location, molecular characteristics, and glioma neuroscience correlative markers will be required to draw conclusions about the potential role of levetiracetam for DMG therapy" (lines 266-269).
The results are novel. The authors give a very thorough description of limitations, particularly of all retrospective analyses.
Overall, if these data are further expanded (as suggested by authors in lines 266-269) and prospectively validated in clinical cohorts, this study will have a high significance because it expands the therapeutic options in an area of very high unmet clinical need by a so far un-acknowledged/neglected mechanism.

We thank the Reviewer for the positive and supportive comments, and helpful suggestions.

II Specific comments

1) Retrospective analyses of three patient cohorts

a) Major comments

- The authors acknowledge the main limitations of these analyses themselves. The retrospective setting is indeed very challenging, particularly if one takes into account that they included patients that had been treated between 1990-2020 (Standford), 2012-2021 (Michigan) and 2006-2021 (Hamburg). It is very demanding to ensure a thorough data collection in a retrospective design and thus this reviewer really acknowledges this effort. How did the authors ensure the collection of a harmonized clinical data set? Did they perform a central pathology review? Did they perform a central imaging review? All these assessments could be performed in a retrospective analyses to increase the comparability of data. Please add information, data and comment.

A team of neuro-oncologists (A.M., D.T., M.M.) systematically reviewed full medical records for each individual patient to confirm diagnosis, survival, treatments, and molecular data if available. During this

review, patient cohort assignment was corrected as needed and those with no follow up medical records available were excluded. Institution-specific radiographic reviews have been conducted at University of Michigan and Stanford University. In response to this important comment, a central pathology review has now been conducted by a board-certified neuro-oncologist (Hannes Vogel) on both Stanford and University of Michigan DMG patients for which tissue samples were available.

- The sample sizes of the three cohorts are very different and it is quite challenging to extract all relevant information from the present manuscript because the information is distributed in the legend and in the text. Please provide a CONSORT-like overview for all three cohorts. Furthermore, the validation cohort is very small, only comprising 19 vs 15 patient as this reviewer understands from Figures 1 e-f. Please expand the number of the validation cohort, e.g. by reaching out to further centers. An increase of sample size for validation plus central review of path/imaging will significantly increase the value of the retrospective analyses and will inform further prospective cohorts.

We have now created a CONSORT-like diagram in response to this helpful comment. This has been added to Figure 1 and is shown below.

a.

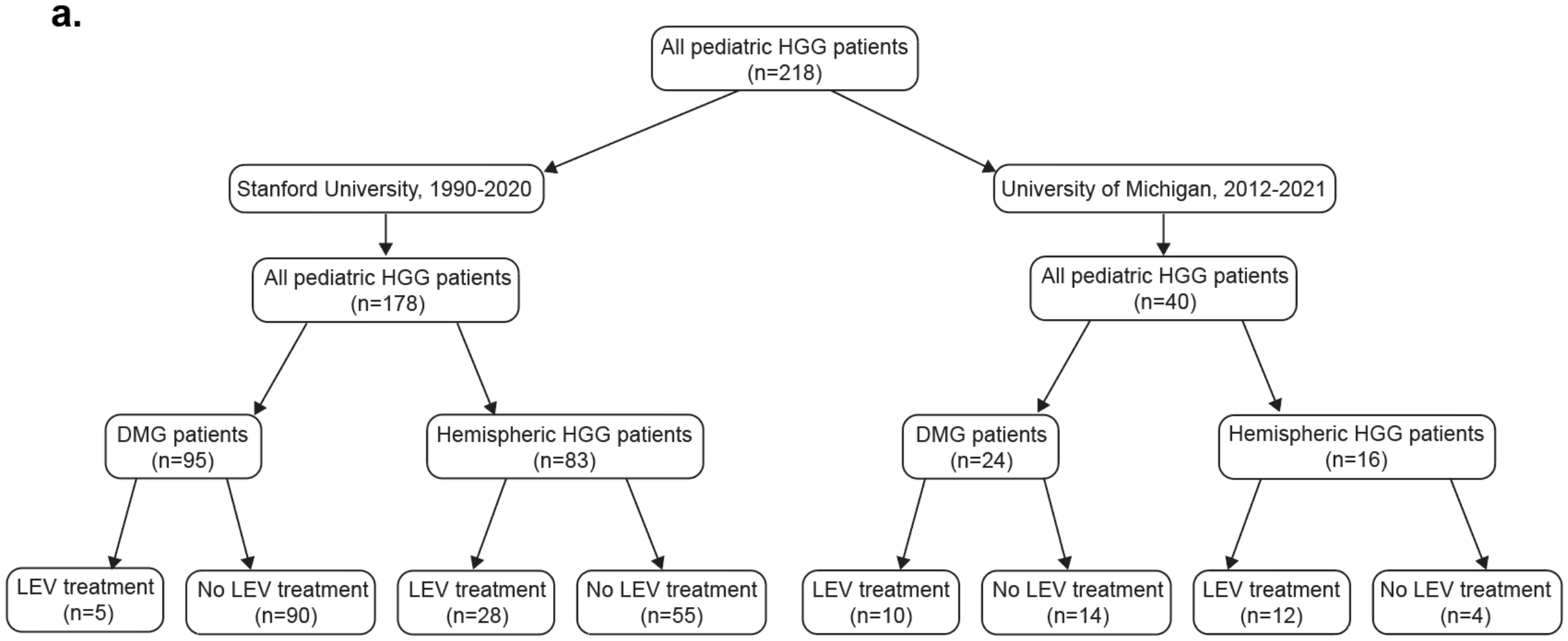

We have also added an additional retrospective patient data from UCSF. Because UCSF has been very involved in clinical studies of ONC201, we included only those patients who were not enrolled on clinical trials to avoid the confound of ONC201-related efficacy. This cohort alone is under-powered on its own but shows the same trend as the other cohorts: an increase in survival in levetiracetam-treated DMG patients. To obtain appropriate power, we combined the two validation cohorts and found a significant increase in survival in patients treated with levetiracetam. As there was a difference in the median overall survival in each validation cohort, we normalized the data to the overall survival of the control group for each cohort. The data, along with the corresponding CONSORT-like diagrams for the validation cohorts, have been added to Extended data figure 1 and are shown below.

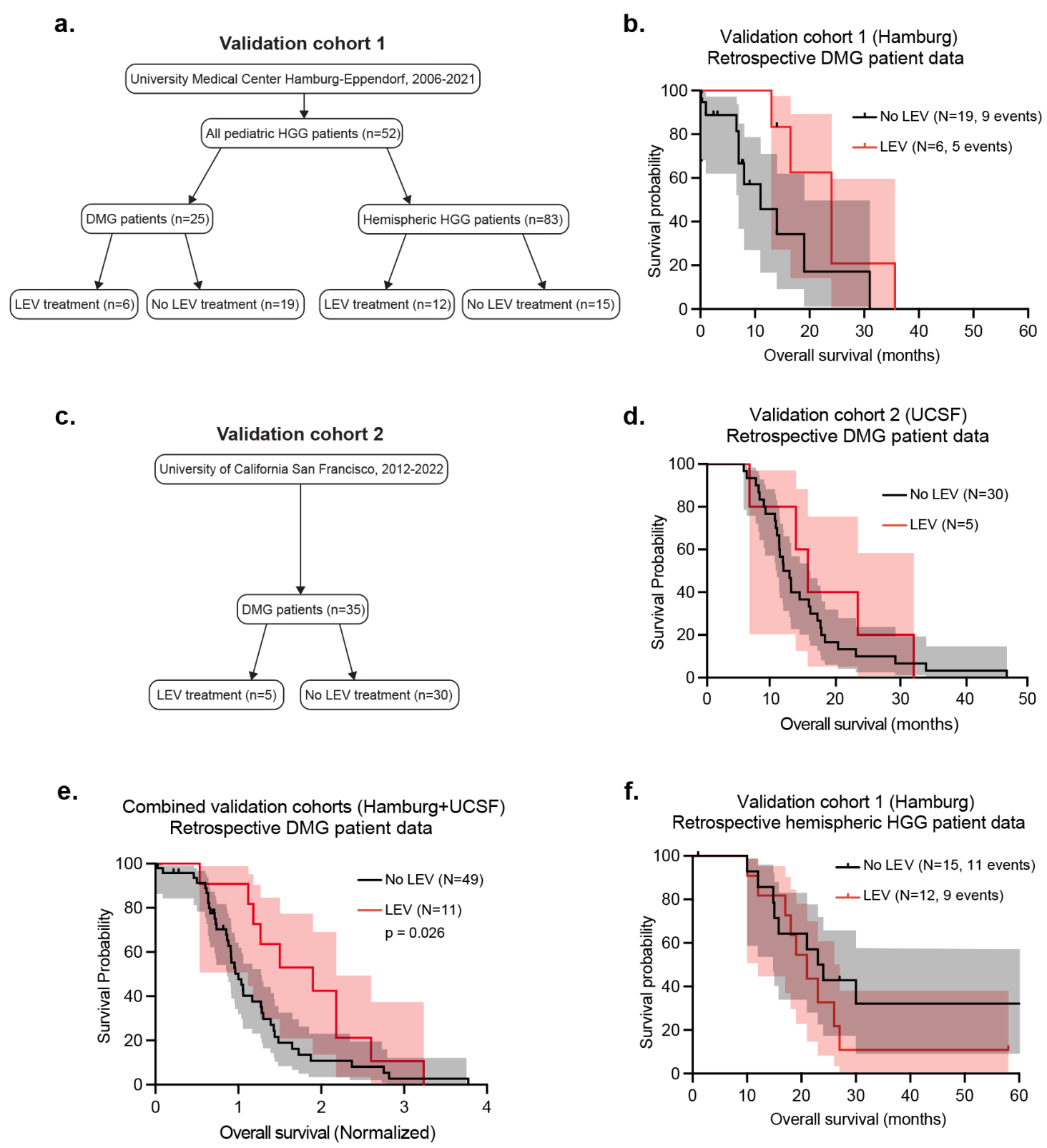

Extended Data Figure 1. Retrospective patient data validation.
a. Overview of patients included in validation cohort 1 from University Medical Center Hamburg-Eppendorf.
b. Validation cohort 1, from University Medical Center Hamburg-Eppendorf, diffuse midline gliomas: Kaplan–Meier overall survival (OS) curves of retrospective data from DMG patients treated with LEV (LEV: median OS = 24.0 months, n = 6; no LEV: median OS= 11.0 months, n = 19).
c. Overview of patients included in validation cohort 2 from University of California San Francisco. Only patients who were not on a clinical trial were included.
d. Validation cohort 2, from University of California San Francisco (UCSF), diffuse midline gliomas: Kaplan–Meier overall survival (OS) curves of retrospective data from DMG patients treated with LEV (LEV: median OS = 15.5 months, n = 5; no LEV: median OS = 12.2 months, n = 30).
e. Combined validation cohorts, diffuse midline gliomas: Kaplan–Meier overall survival (OS) curves of retrospective data from DMG patients treated with LEV, normalized to median of no LEV control for each dataset (LEV: normalized median = 1.89785, n = 11; no LEV: normalized median = 1, n = 49), two-tailed Gehan-Breslow-Wilcoxon test.
f. Validation cohort 1, hemispheric high-grade glioma: Kaplan–Meier overall survival (OS) curves of retrospective data from hemispheric high-grade glioma (HGG) patients treated with LEV (LEV: median OS = 21.0 months, n = 12; no LEV: median OS= 23.5 months, n = 15), two-tailed log rank test.

b) Minor comments

- Lines 292-296: The authors comment on IRB approvals for Stanford and Michigan. Please include information on Hamburg.

We have now added description of IRB approval information for Hamburg as well as the new UCSF cohort to the Methods section.

2) Preclinical experiments

a) Major comments

- The authors present data from patient-derived xenograft models. These models are highly informative and helpful to investigate proof-of-concepts and also to investigate human tumors in vivo. As the effect of levetiracetam and basically any other anti-seizure medication is mainly on the brain microenvironment (i.e.: tumor host interaction) and not a pure cell-intrinsic effect, this reviewer recommends further experiments in syngeneic settings for their analyses, e.g. syngeneic murine models and organoid/microtumors ex vivo. Please add experimental data.

We thank the reviewer for this suggestion. As neuron-to-glioma synapses form in patient-derived xenografts, these are useful models for studying tumor-(neuronal) microenvironment interactions. To confirm these findings in an immunocompetent model, we have now examined the effect of levetiracetam treatment on DMG proliferation and mouse survival in a syngeneic, immunocompetent murine model of DMG (the MADR *in utero* electroporation model of DMG, allografted to the pons). Like the human DMG models, we found reduced DMG cell proliferation in mice treated with levetiracetam. These data have now been added to Figure 2 and are shown below.

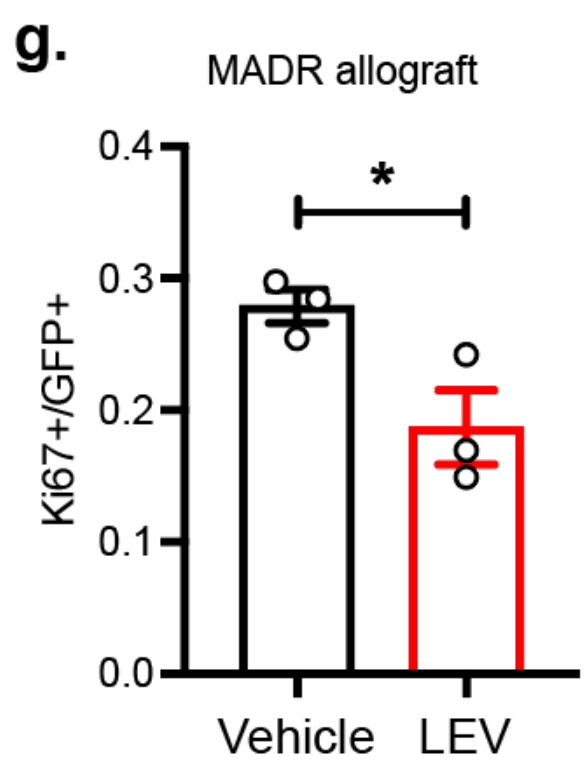

- In Section Results and in Figure 2, the authors present survival data from murine xenograft models in panels a, b. Afterwards, they focus on showing effects on proliferation (2c-j) and do not show further survival effects. Furthermore, this reviewer does not see much on the provided "representative confocal images". Please add more survival data, histology data and more assessable images on tumor histology and immunohistochemistry. Did the authors observe any effect on tumor volume?

We have now added a second xenograft model for mouse survival studies (SU-DIPG-36), also demonstrating extended survival in xenografted mice treated with levetiracetam, shown in Figure 2b and below. We have also now added more histological analyses demonstrating a decrease in tumor burden in mice treated with levetiracetam. We appreciate the suggestion for more accessible images and have included high-level imaging to view the tumor within the entire brain slice, which was used for the tumor burden analysis. These data have been included in Figure 2 and are shown below.

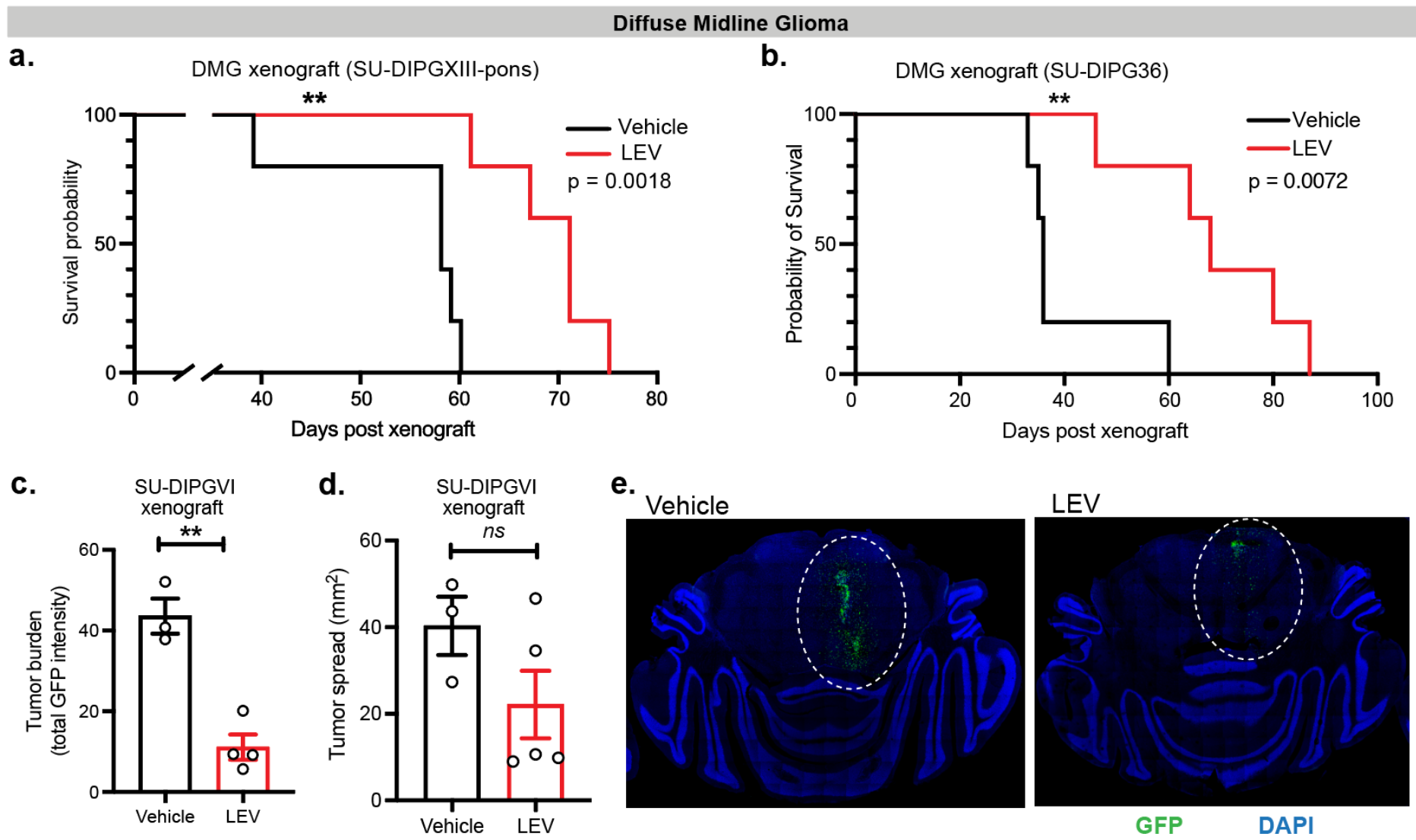

Figure 2. Levetiracetam extends survival and reduces glioma growth in DMG but not hemispheric HGG preclinical models.
a-b. Kaplan–Meier survival curves of mice with orthotopically xenografted SU-DIPG-XIII-pons (**a.**) or SU-DIPG36 (**b.**) tumors treated with levetiracetam (LEV) or vehicle (n = 5 mice per group).
c-d. Effect of LEV on overall tumor burden (**c.**) and tumor spread (**d.**) in mice with patient-derived DMG xenografts (SU-DIPGVI; vehicle, n = 3; LEV, n = 5), two-tailed Student's t-test.
e. Representative confocal images of xenografted SU-DIPGVI cells (green) in the pons of mice treated with vehicle or LEV. White dotted line represents area of tumor spread.

b) Minor comments

- The number of mice per experimental group varies between the different experiments that are outlined in Figures 2 and 3. Please add the corresponding number in the figure to allow a better and quicker overview at one glance.
Data points on all graphs represent one mouse, well or cell, and are detailed for each figure panel in the figure legends.

3) Tumour subtype-specific mechanism of brain cancer neurophysiology: Effects of levetiracetam on GABAergic neuron-to-glioma versus neuron-to-neuron synaptic currents in DMGs

- The experiments presented in Section Results lines 185-228 and in Figure 4 aim at identifying the mechanism of action of levetiracetam on DMG. Using brivaracetam (a more selectively acting anti-seizure medication, i.e. compared with levetiracetam more SV2A-specificity), they conclude that levetiracetam does not act by inhibiting SV2A because they do not observe any effect with brivaracetam. Yet, the dosages that the authors used here for both drugs might effect these different phenotypes, too. Please comment and explain the chosen dosage and its relevance compared with the clinical setting. Furthermore, it would be necessary to perform additional genetic validation by SV2A silencing.

We thank the reviewer for raising this important point. The effect of LEV on high frequency postsynaptic currents in neurons is observed at 100um (Meehan et al 2012, Yang et al., 2007), which is why we used this concentration here. As BRV is a much more potent drug, it has a greater effect on high frequency postsynaptic currents at lower concentrations as compared to LEV (Yang et al., 2015). We have added the explanation for the doses in the manuscript: "The potency of brivaracetam is high, such that it has a

greater effect on high frequency postsynaptic currents at lower concentrations (Yang et al., 2015) as compared to levetiracetam, for which the effect on high frequency postsynaptic currents in neurons is observed at 100 µM (Meehan et al 2012; Yang et al., 2007)."

We have now added experiments with genetic knockdown of SV2A. We performed an shRNA knockdown of SV2A in human induced pluripotent stem cell-derived GABAergic neurons, cultured them alongside patient-derived DMG cells, and assessed proliferation of the glioma cells after 48 hours. Our previous studies have demonstrated an increase in proliferation of DMG cells when co-cultured with mixed (glutamatergic and GABAergic) cortical neurons compared to glioma monoculture (Venkatesh et al., 2019 *Nature*; Taylor et al., 2023 *Nature*). Glioma cells co-cultured with hiPSC-derived GABAergic neurons exhibited increased proliferation compared to glioma monoculture. Levetiracetam decreased proliferation of glioma cells co-cultured with GABAergic neurons, as expected, and there was no effect of SV2A knockdown in GABAergic neurons on the anti-proliferative effect of levetiracetam. These data have been added to Figure 4 and Extended Data Figure 3, and are shown below:

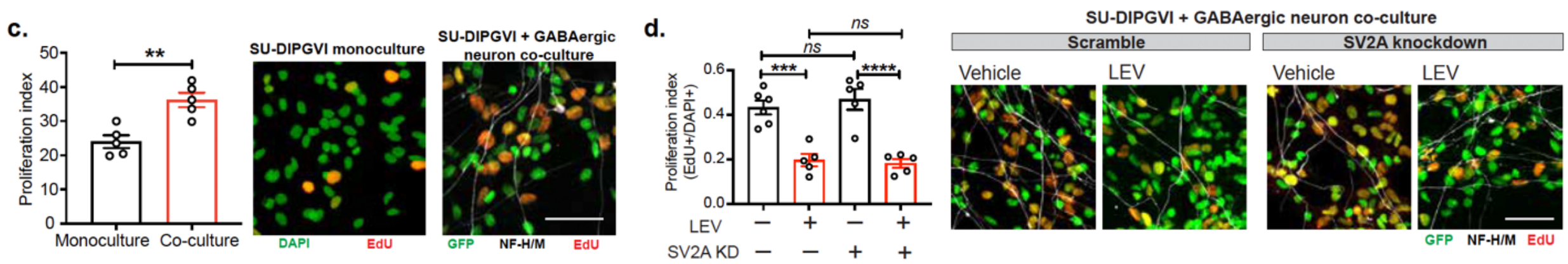

Figure 4. Levetiracetam selectively reduces GABAergic neuron-to-glioma synaptic currents.
c. Quantification of DMG cell proliferation (EdU^+/GFP^+) in monocultures or co-culture with GABAergic neurons. Unpaired, two-tailed Welch's t-test. Right, confocal micrographs showing proliferating DMG cells in monoculture or in co-culture with GABAergic neurons. EdU: red, NF-H/M: white, DAPI: green (left image), GFP: green (right image). Scale bar, 50 µm.
d. Quantification of DMG cell proliferation (EdU^+/GFP^+) in GABAergic neuron co-cultures transduced with scrambled or SV2A-targeting shRNA and treated with vehicle or LEV. One-way ANOVA with Tukey's post hoc test. Right, confocal micrographs showing proliferation (EdU, red) of DMG cells expressing GFP (green) co-cultured with GABAergic neurons expressing NF-H/M (white). Scale bar, 50 µm.

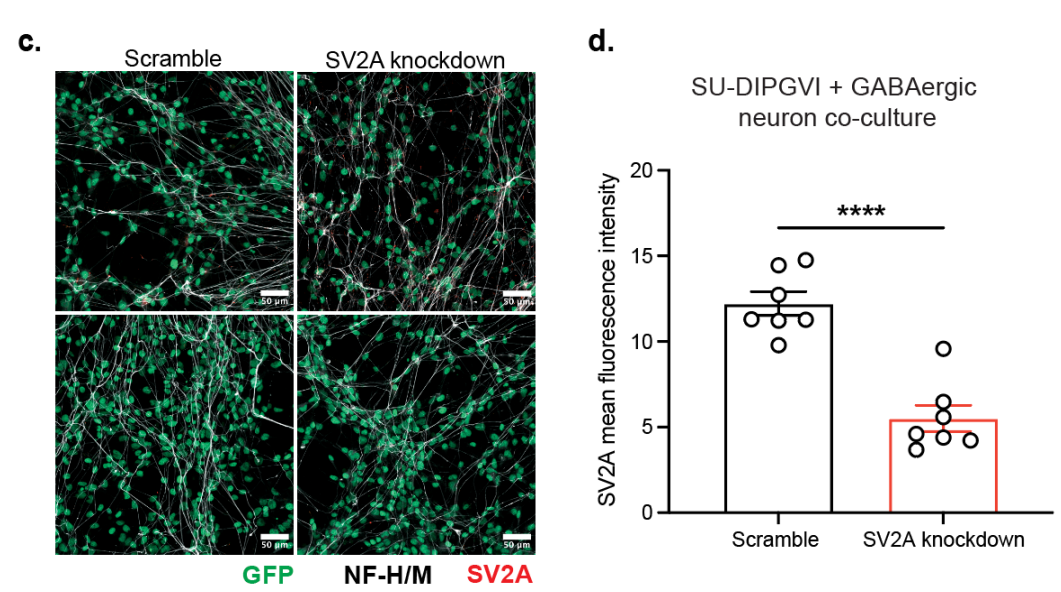

Extended Data Figure 3. Confirmation of glioma patch and SV2A knockdown.
c. Confocal micrographs showing SV2A expression in GABAergic neuron-glioma co-cultures following scrambled shRNA (top) or SV2A knockdown (bottom) neuronal transduction. GFP: green, SV2A: red, NF-H/M: white. Scale bars, 50 µm.
d. Quantification of mean fluorescence intensity of SV2A signal in GABAergic neurons transduced with scrambled or SV2A-targeting shRNA. Unpaired, two-tailed Welch's t-test; ****$p < 0.0001$. Data represent mean ± SEM.

Reviewer #4 (Remarks to the Author):

In this manuscript, the authors report that the approved antiepileptic drug levetiracetam has specific anti-tumor effects in DMG, specific both with respect to tumor entity and antiepileptic drug. This is supported by retrospective patient cohorts from 3 institutions, and two mouse models of DMG. Finally the authors

demonstrate data that speaks for a specific influence of LEV on the GABA receptor via a mechanism that still needs to be explored.

This study adds significantly to our understanding of how distinct drugs interfering with neuronal activity can selective influence neuron-to-cancer communications. The authors showed before that GABA-ergic antiepileptic drugs should be avoided, because they fuel tumor growth in DMG; they now demonstrate a tumor cell-specific GABA-inhibitory effect of LEV which is most likely responsible for the antitumor effects (mouse survival and tumor cell proliferaion). The translational / clinical relevance is strong.

The manuscript is very clearly and well written, and the data is presented in a thoughtful and critical way. The methods and statistics appear all adequate. I could find no errors.
We thank the Reviewer for the positive and supportive comments.
Two points should be addressed:

1. Fig. 4: how sure can one be that GABA-ergic synaptic currents have been recorded in the tumor cells?

This is a very important point, and we thank the reviewer for commenting on this. The engrafted tumor cells express GFP to aid in visualization for patch clamp recording from malignant cells. During recording, cells are filled with Alexa 568 fluorescent dye to validate in real-time that the GFP+ tumor cell was indeed the cell being recorded. This approach is detailed in our previous papers (Venkatesh et al., 2019, *Nature*; Taylor et al., 2023, *Nature*; Barron et al., 2025 *Nature*), and we have added more description of this method to this manuscript and provided an example of a dye-filled glioma cell in Extended Data Figure 3, also shown below.

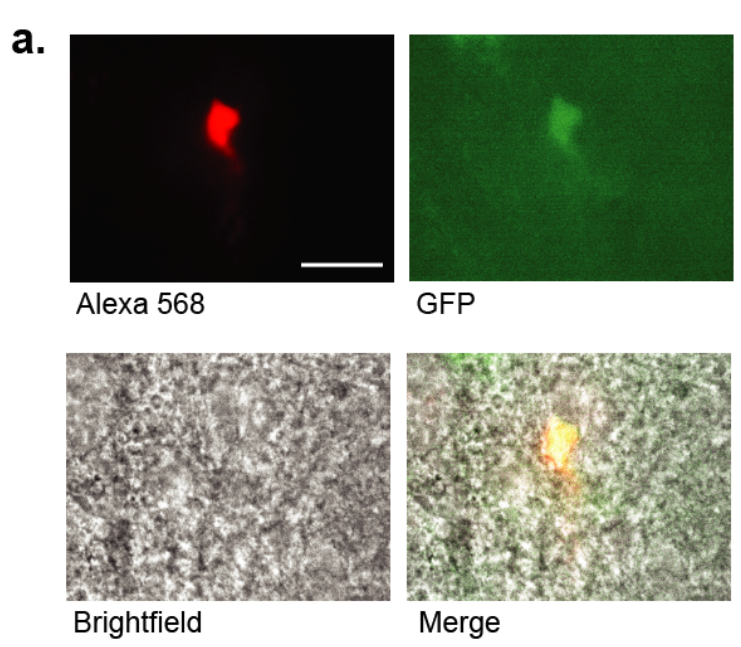

Extended Data Figure 3. Confirmation of glioma patch and SV2A knockdown.
a. Fluorescent and brightfield microscopy images showing a GFP+ glioma cell (green) dye-filled during patch-clamp recording (Alexas 568, red) and visible with brightfield imaging. Scale bar, 50 µm.

We also validate that each recorded current is mediated by the neurotransmitter or receptor expected by adding an inhibitor at the end of the recording. Picrotoxin, a $GABA_A$ receptor antagonist, was used to inhibit the remainder of the GABAergic current after levetiracetam perfusion. A representative trace of this has now been included in Figure 4a, as shown below:

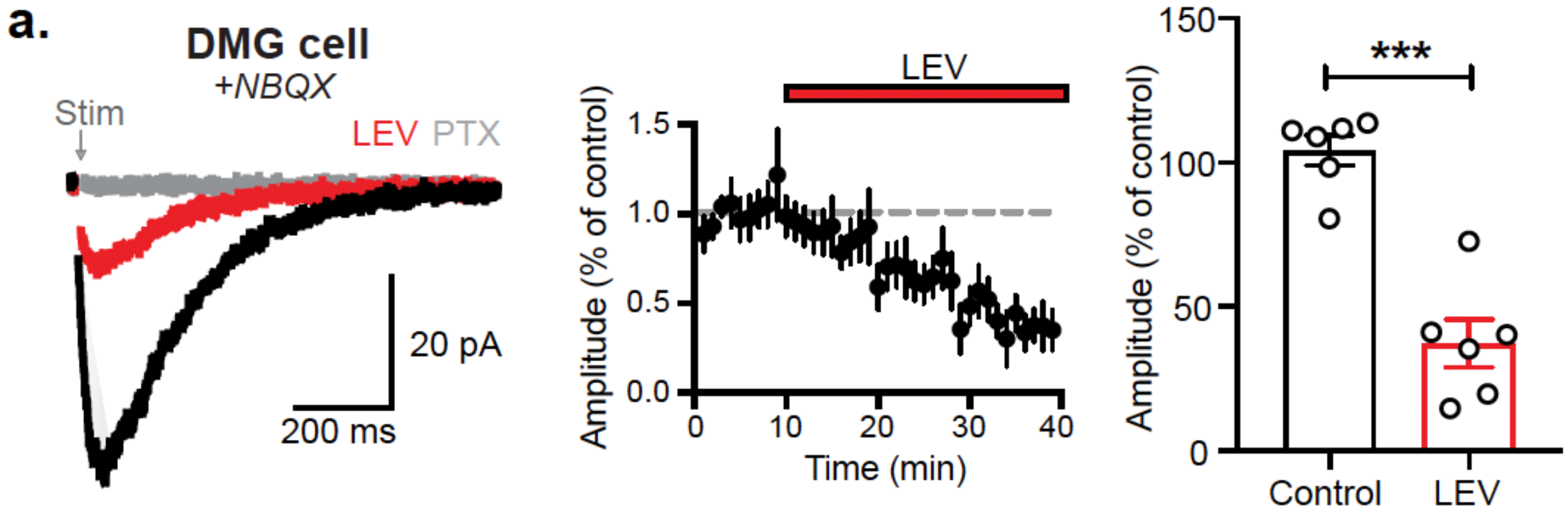

Figure 4. Levetiracetam selectively reduces GABAergic neuron-to-glioma synaptic currents.

a. Left, representative averaged trace of GABAergic postsynaptic currents (PSCs) in DMG before and after 30-minute perfusion of 100 µM levetiracetam (LEV), recorded in the presence of NBQX to block AMPA receptor-mediated currents. Center, time course of GABAergic PSC amplitude attenuation in response to LEV. Right, quantification of GABAergic PSC amplitude in DMG before and after 30-minute LEV perfusion as % of control (n = 6 cells from 5 mice), paired Student's t-test.

Can the authors modulate the recorded currents in control tumor cells treated with NBQX when a GABA modulator is added?

Yes. In addition to the PTX inhibition of GABAergic currents, we have demonstrated in a previous study that these GABA currents in glioma cells (recorded in the presence of NBQX) are sensitive to the benzodiazepine lorazepam, a positive allosteric modulator of $GABA_A$ receptors. These data can be seen in Barron et al., 2025, *Nature*, and are replicated below for ease of reference.

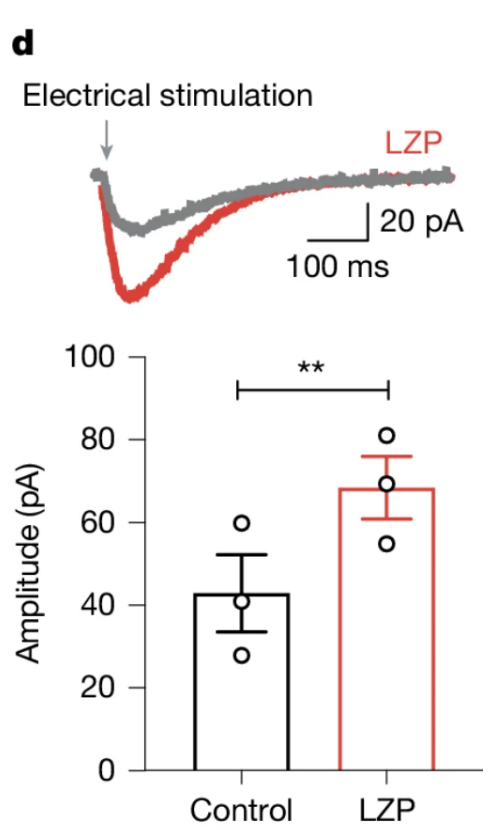

Barron et al, 2025 Nature Figure 4d. Representative trace of GABAergic postsynaptic current in DMG elicited by electrical stimulation in the presence of NBQX before and after perfusion of 10 µM lorazepam (LZP). Below, quantification of current amplitude (n=3 cells from 3 mice, p=0.0386), two-tailed paired Student's t-test.

2. It would be a strong addition to the story if the authors can provide more data on the molecular mechanism of what LEV is exactly modulating in the DMG cells. Can the authors provide gene expression and/or proteomics data? How is GABA signaling exactly influenced (molecularly / electrophysiologically)? Brivaracetam is a much more specific SV2A inhibitor than LEV, implying the

possibility that other neurotransmitter receptors or neuronal factors might play in role on the neuronal side.

This is an important question that will take several years to answer. The targets of LEV in neurons have been studied for decades and are still incompletely understood. The effect is too quick (minutes) to be explained by gene expression changes, so effects on protein trafficking or post-translational modifications are more likely. Given that this is a cancer-specific, SV2A-independent mechanism, it will require likely years to figure out. First author Tara Barron plans to tackle this question of the mechanisms of levetiracetam action at DMG GABAergic synapses in her new independent faculty laboratory at UT Southwestern in the coming years. While answering this enigmatic mechanism is beyond the scope of the present study, we have added discussion of this important question and the need to answer it in future work:

"Levetiracetam specifically attenuates GABAergic neuron-to-glioma synaptic currents through a tumor-selective, SV2A-independent mechanism. Future work will be required to determine this tumor-selective mechanism of levetiracetam action."

Point-by-point response to reviewer comments

Reviewer #1:
Remarks to the Author:
No further comments or suggestions
We thank the reviewer for the positive comments.

Reviewer #2:
Remarks to the Author:
The authors present a revised version of their study.
They have added new data including a new validation cohort, central neuropath review, a CONSORT chart, extensive further preclinical experiments.
All questions of this reviewer have been addressed.
We thank the reviewer for the positive comments.

The clinical data derived from retrospective data sets comprise a rather small set of patients with Diffuse Midline Glioma (DMG) and LEV treatment (n=5 in the validation cohort from Hamburg/Germany, and n=5 from the UCSF validation cohort).
How do the authors judge the relevance of clinical data and comparisons of LEV-treated DMG versus non-LEV-treated with these rather small case numbers?
We appreciate this point and have highlighted the caveat of small patient numbers in the text. We are reassured by the fact that the Hamburg and UCSF validation cohorts support the larger Stanford-Michigan discovery cohort (n = 119 DMG patients) findings, with each cohort showing longer survival for DMG patients taking levetiracetam.

Reviewer #4:
Remarks to the Author:
The authors have responded well to my comments.
We thank the reviewer for the positive comments.
